# The Intersection of Genetics and Neuroimaging: A Systematic Review of Imaging Genetics in Neurological Disease for Personalized Treatment

**DOI:** 10.1007/s12031-025-02350-7

**Published:** 2025-05-13

**Authors:** Mahinaz A. Mashhour, Ibrahim Youssef, Manal Abdel Wahed, Mai S. Mabrouk

**Affiliations:** 1 Biomedical Engineering Department, Misr University for Science and Technology, Giza, Egypt; 2 Systems and Biomedical Engineering Department, Cairo University, Giza, Egypt; 3 Center for Informatics Science (CIS), School of Information Technology and Computer Science, Nile University, Giza, Egypt

**Keywords:** Precision medicine, Imaging genetics, Radiogenomics, Neurological diseases, Voxel-based morphometry

## Abstract

Imaging genetics is one of the important keys to precision medicine that leads to personalized treatment based on a patient’s genetics, phenotype, or psychosocial characteristics. It deepens the understanding of the mechanisms through which genetic variations contribute to neurological and psychiatric disorders. This systematic review overviews the methods and applications of imaging genetics in the context of neurological diseases, mentioning its potential role in personalized medicine. Following PRISMA guidelines, this review systematically analyzes 28 studies integrating genetic and neuroimaging data to explore disease mechanisms and their implications for precision medicine. Selected research included multiple neurological disorders, including frontotemporal dementia, Alzheimer’s disease, bipolar disorder, schizophrenia, Parkinson’s disease, and others. Voxel-based morphometry was the most common imaging technique, while frequently examined genetic variants included APOE, C9orf72, MAPT, GRN, COMT, and BDNF. Associations between these variants and regional gray matter loss (e.g., frontal, temporal, or subcortical regions) suggest that genetic risk factors play a key role in disease pathophysiology. Integrating genetic and neuroimaging analyses enhances our understanding of disease mechanisms and supports advancements in precision medicine.

## Introduction

Precision medicine (PM) customizes diagnostics and treatments to an individual’s genetic, biomarker, phenotypic, and psychosocial characteristics, moving beyond the generalized approach of traditional medicine (Pinker et al. 2018). It relies on methods such as imaging genetics, which merges genomic data such as genome-wide association studies (GWAS) (Bogdan et al. 2017; Elliott et al. 2018) with neuroimaging such as positron emission tomography (PET) or magnetic resonance imaging (MRI) to discover how gene variants influence brain structure and function (Chen and Coppola 2015; Ameis and Szatmari 2012; Lorenzi and Altmann 2024; Zhang et al. 2024). Understanding these specific relationships between genes and their influence on the brain reveals how different neural pathways cause each behavior, cognitive trait, or predisposition to neurologic and mental diseases (Glahn, et al. 2015). However, despite advances in imaging genetics, important gaps remain. First, the heterogeneity of imaging techniques and genetic analyses complicates direct comparisons across studies. Second, many imaging-genetics studies rely on relatively small sample sizes, limiting generalizability. Finally, questions remain about how these findings can be translated into clinical practice to achieve truly personalized treatments. This review addresses these gaps by systematically analyzing how imaging genetics is applied across multiple neurological conditions.

Imaging genetics has already shown promise in multiple neurological conditions, including Alzheimer’s disease (AD) (Huang et al. Mar. 2016) (Gomar et al. 2016) (Hibar et al. 2015), schizophrenia (Cousijn et al. 2014) (Kalmady et al. 2014), autism spectrum disorders ((Martin-Brevet et al. 2018; Nisar and Haris 2023), and major depressive disorder (Dannlowski et al. 2015). Large consortia—such as ENIGMA (Thompson et al. 2014) and IMAGEN (Schumann et al. 2010)—have supported extensive research and findings in this domain. These efforts are critical for understanding how genetic factors drive structural and functional brain changes, ultimately informing personalized interventions.

**Fig. 1:**
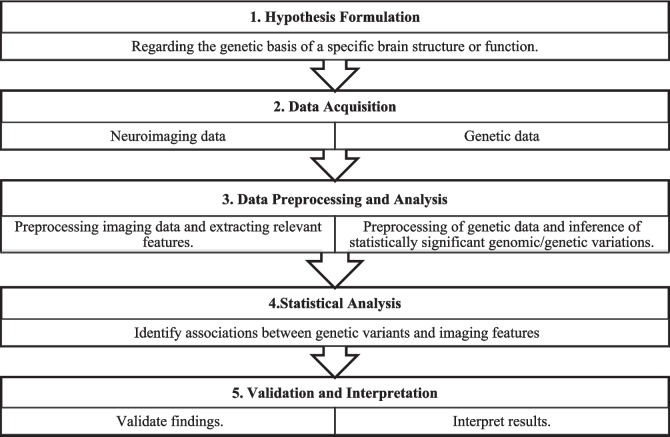
The multi-step approach for imaging genetics research

The multi-step approach for imaging genetics research is shown in Fig. 1. The first step is the hypothesis formulation that should include the genetic variations and brain imaging phenotypes of interest, followed by imaging and genetic data acquisition. Then, imaging data is processed to extract features like brain volume, and genetic data is processed to evaluate genetic variations based on the phenotype under investigation. Statistical methods are then used to evaluate the relationship between brain features and genetic variations. Finally, validation and interpretation take place to gain a better understanding of the results.

The process of imaging genetics includes five key steps: formulating a hypothesis, acquiring data, preprocessing and analyzing data, conducting statistical analysis, and validating results.

### Hypothesis Formulation

Hypothesis formulation in imaging genetics is generally based on the idea that changes in genome structure or gene activity may affect changes in brain structure, function, or connectivity. A well-phrased hypothesis of imaging genetics ought to spell out a specific and testable association between the genetic factors and the brain imaging outcome that would offer insight into the biological pathways underlying a neurological or psychiatric disorder. It starts with identifying those genetic variants—for example, SNPs, which are mutations in gene sequences deemed to affect neurological disorders, neurodegenerative diseases, or cognitive traits. The type of neuroimaging also comes into relevance: structural MRI for assessing atrophy of the brain, functional MRI to investigate activity of neural systems, PET-MRI to measure atrophy and metabolic dysfunction, or MRI-based diffusion tensor imaging in order to look at white matter integrity. Then, a hypothesis is formulated through the proposing of how particular genetic variants relate to measurable changes in these brain regions or pathways.

### Data Acquisition

Imaging genetics studies utilize large-scale datasets such as UK Biobank, which provides genotype data and MRI/functional MRI (fMRI) from half a million UK participants (Sudlow et al.^2015^). The Human Connectome Project offers detailed neural pathway maps through structural, functional, and diffusion MRI, alongside genetic data (Essen et al. 2012). The Adolescent Brain Cognitive Development (ABCD) Study collects comprehensive neuroimaging and genetic data from thousands of children to study brain development (Barch et al. 2018). The ENIGMA Consortium (Thompson et al. 2014) collates global data on brain structure and disease, while the IMAGEN Study (Schumann et al. 2010) investigates genetic effects mediated by environmental factors on adolescent brain development. The National Institute of Mental Health (NIMH) (“National Institute of Mental Health. (n.d.)) data archive hosts data from various mental health studies, and the Alzheimer’s Disease Neuroimaging Initiative (ADNI) (Mueller et al. 2005) aims to identify Alzheimer’s disease (AD) biomarkers. The Parkinson’s Progression Markers Initiative (PPMI) is a large study designed to find progression markers for the different dimensions of Parkinson’s disease (PD) that integrate genetic and imaging data (Marek et al. 2011). Lastly, the Psychiatric Genomics Consortium (PGC) studies genetic contributions to psychiatric disorders, and one main aspect is the imaging data (Psychiatric Genomics Consortium (n.d.)).

### Data Preprocessing and Analysis

The preprocessing and analysis phase ensures that results are accurate, true, and interpretable. Considering the high dimensionality and complexity of the two types of data, their preprocessing is highly necessary for noise reduction and error correction.

#### Image Processing and Feature Extraction

For neuroimaging data, this involves spatial normalization to align images from different subjects into a common space, segmentation to identify and isolate different brain regions, smoothing and filtering to enhance the signal-to-noise ratio in the images, and feature extraction, such as brain volumes or functional connectivity (Ashburner and Friston 2000). For spatial preprocessing, statistical analysis, and visualization of brain imaging data sequences from MRI/fMRI and PET, one of the main tools used is statistical parametric mapping (SPM) (Ashburner and Friston 2000; Yao et al. 2020; Du et al. 2023). Another widely used tool is the FMRIB Software Library (FSL) (Jenkinson et al. 2012; Viar-Hernández et al. 2024), which offers a full suite for the analysis of fMRI, MRI, and diffusion tensor imaging (DTI) data, including preprocessing, registration, segmentation, and statistical analysis. Reconstructing and statistically analyzing cortical surfaces are facilitated by the automated measurement of brain volumes and cortical thickness, which is made possible by FreeSurfer (Fischl 2012; Beaulac et al. 2023). With the purpose of analyzing and displaying fMRI data, Analysis of Functional NeuroImages (AFNI) (Cox 1996) (Anderson et al. Jun. 2020) provides a range of programs that meet different requirements related to statistical modeling and preprocessing. Advanced Normalization Tools (ANTs) (Avants et al. Feb. 2011) (Cataldo-Ramirez et al. Jul. 2023) stand out for image registration and normalization due to their high-dimensional registration capabilities, which are essential for tasks like segmentation and cortical thickness mapping. To improve the ability to analyze cortical structures, BrainSuite (Shattuck and Leahy 2002) (Wegdan et al. 2023) offers specialized tools for cortical surface extraction and brain image segmentation. The open-source Neuroimaging in Python (NiPy) library (Millman and Brett 2007) provides a wide range of statistical analysis, data manipulation, and visualization features to facilitate the analysis of structural and functional neuroimaging data. Finally, WebMRI (Harmouche et al. 2023) grants access to MRI data through a web interface, providing a user-friendly environment for handling and manipulating MRI data. By facilitating the preprocessing, analysis, and visualization of intricate neuroimaging data, these tools help researchers advance the integration of genetic and imaging datasets in imaging genetics studies.

#### Genetic Data Analysis

Quality control involves checking genetic data for errors and ensuring its accuracy to maintain data integrity. First, the genotyping determines the genetic makeup of individuals, and imputation infers missing genotypes to complete the dataset. The next critical phase is statistical analysis, where techniques such as GWAS or candidate gene approaches are employed to identify genetic variants associated with specific brain features. Tools used for genetic data preprocessing include PLINK (Purcell et al. 2007; Zhang et al. 2021; Karkar et al. 2021), a fundamental toolset for whole-genome association and population-based linkage analyses, providing capabilities for quality control, association testing, and data management. Genome-wide Complex Trait Analysis (GCTA) ((Yang et al. 2011; Gao et al. 2021)) aids in estimating the proportion of phenotypic variance explained by genetic variants and performing association analyses to understand the genetic architecture of complex traits. For association testing, SNPTEST (Marchini et al. 2007) performs both frequentist and Bayesian tests, handling imputed genotype data to enhance the accuracy of genetic association studies. IMPUTE (Howie et al. 2009) is critical for predicting missing genotypes using haplotype information from a reference panel. Finally, Multi-marker Analysis of Genomic Annotation (MAGMA) (Leeuw et al. 2015) (Bharthur Sanjay, et al. 2022) helps in gene/pathway analysis to associate genetic data with complex traits.

### Statistical Analysis

A variety of statistical methods are used to analyze the complex relationship between genetic variations and brain structure. Statistical analysis methods such as analysis of variance (ANOVA) and *t*-tests are used to compare brain imaging metrics between different genetic groups for significant differences. Multiple regression analysis can be used to evaluate the effect of different sets of genetic variants on the brain, and multivariate analysis of covariance (MANCOVA) considers the differences across many brain measures at once (Teipel et al. 2007). Finally, general linear models (GLM) (Ashburner and Friston 2000) are employed to model the relationship between genetic variants and brain features.

### Validation and Interpretation

An important part to prove the reliability and reproducibility of results is validation, wherein associations discovered are reproduced in independent datasets to assess their robustness. By combining the results of multiple studies, meta-analysis enhances statistical power and allows researchers to characterize the magnitude and direction of genetic effects in different populations, thereby increasing the ability to interpret findings from complex analyses between genetics data (i.e.., single or multi-locus) with various brain imaging phenotypes.

In this research, a comprehensive systematic literature review of imaging genetics research concerning neurological diseases, specifically in the last 10 years, is introduced. In the following sections, we will dig deeper into the methodologies and applications of imaging genetics, demonstrating its transformational promise for expanding our understanding of brain disorders and bringing in a new era of personalized medicine.

## Methods

### Protocol

A thorough examination of previous studies was carried out in accordance with the accepted Preferred Reporting Items for Systematic Reviews and Meta-Analyses (PRISMA) (Wells, et al. 2025) in order to guarantee transparency.

### Search Strategy

A focused literature search was conducted to evaluate recent advancements in imaging genetics, using the PubMed database due to its extensive coverage of biomedical, neuroimaging, and neuroscience research. To maintain focus, methodological consistency, and relevance to the biomedical scope of this review, additional searches in databases, as well as manual reference checks, were not performed. The final search was completed on October 14, 2024. The search terms were selected to capture studies that included both morphological measurements of the central nervous system (e.g., “morphometry,” “voxel-based morphometry”) and genetic variation (“genetic variation,” “genetic variations”). We combined these with “central nervous system” to limit results to neurological or neuropsychiatric contexts. To maximize retrieval accuracy, Boolean operators and standardized MeSH terms were applied. We used combinations of the following terms: ((“morphometries”[All Fields] OR “morphometry”[All Fields]) AND (“central nervous system”[MeSH Terms] OR (“central”[All Fields] AND “nervous”[All Fields] AND “system”[All Fields]) OR “central nervous system”[All Fields]) AND (“genetic variation”[MeSH Terms] OR (“genetic”[All Fields] AND “variation”[All Fields]) OR “genetic variation”[All Fields] OR (“genetic”[All Fields] AND “variations”[All Fields]) OR “genetic variations”[All Fields])) AND ((y_10[Filter]) AND (ffrft[Filter]) AND (excludepreprints[Filter]) AND (humans[Filter]) AND (data[Filter]) AND (english[Filter]).

### Eligibility Criteria

The inclusion criteria limited studies to English-language, full-text, peer-reviewed articles, published within the last 10 years, based on human data, and excluded preprints. While this approach ensured methodological accuracy, it may have introduced language and publication bias, potentially excluding relevant studies from non-English sources or recent preprint findings. Articles that meet the following inclusion criteria were selected:

1. Study type: original research articles
2. Population: studies focusing on human subjects with a specified neurological or psychiatric condition
3. Genetic data: must include a genetic factor (e.g., SNP analysis, GWAS data, candidate gene approach)
4. Neuroimaging data: must contain MRI, PET, or other structural/functional imaging data
5. Study outcomes: must investigate associations between genetic variants and brain structure/function

### Quality Assessment

Cross-sectional, cohort, and case–control studies were included. Cross-sectional studies capture exposure and outcomes at a single time point, cohort studies follow participants longitudinally to track outcome development, and case–control studies compare individuals with a condition (cases) to similar individuals without it (controls). The critical appraisal tool to assess the quality of cross-sectional studies (AXIS) (Downes et al. 2016) was used to evaluate the methodological quality of the included cross-sectional studies, while the Newcastle–Ottawa scale (NOS) was used to evaluate the cohort and case–control studies (Wells, et al. 2025). Discrepancies in data extraction and quality assessment were resolved through discussion among reviewers. If full consensus was not reached, a majority decision was adopted after group deliberation. To visually summarize the quality assessment results, heatmaps were generated for each study design in Figs. 4, 5, and 6.

### Data Synthesis

Studies were assessed for eligibility based on predefined inclusion criteria, and relevant data were extracted for qualitative synthesis. Due to heterogeneity in study designs, sample characteristics, imaging modalities, genetic variants, and statistical methods, a narrative synthesis was performed instead of a meta-analysis. Key findings were summarized in Table 1, and any discrepancies in data interpretation were resolved through discussion among reviewers to ensure accuracy and consistency.

**Table 1:** Methods and findings in the selected studies

Authors	Trait	Genetic data	Sample size	Ancestry	Statistical methods	Findings
(Sellami, et al. 2018)	Frontotemporal dementia	Mutations in *C9orf72*, *GRN*, and *MAPT* genes	167 participants • 75 carried GRN mutations • 60 carried C9orf72 • 32 carried MAPT mutations	multiple European and North American sites	• Multiple regression model	• The C9orf72 group had left frontal cortical atrophy • The MAPT group had cerebellar atrophy • GRN group had GM reduction in the default-mode network’s posterior structures
(Cash et al. 2018)	Frontotemporal dementia	Mutations in *C9orf72*, *GRN*, and *MAPT* genes	319 participants • 144 non-mutation carriers • 128 presymptomatic mutation carriers • 47 clinically affected mutation carriers (C9orf72, GRN, or MAPT)	13 research centers across multiple countries	• GLM	• *C9orf72 group showed GM reduction in thalamus and cerebellum* • *MAPT group showed GM reduction in the anterior and medial temporal lobes* • *GRN group showed GM reduction in striatum, posterior frontal, and parietal lobes*
(Whitwell et al. 2015)	Frontotemporal dementia	Mutations in *C9orf72*, *GRN*, and *MAPT* genes	58 participants • 11 with C9orf72 • 11 with GRN • 21 with MAPT • 15 sporadic cases	Not specified	*t*-tests	• GRN group showed the fastest whole brain atrophy rates and the highest regional atrophy across all lobes • Sporadic FTD demonstrated more atrophy in the anterior cingulate than both *C9ORF72* and* MAPT* • *C9ORF72* demonstrated more reduction in the left cerebellum and right occipital lobe than did *MAP*
(Luis et al. 2016)	Frontotemporal Dementia	SQSTM1 gene mutations	50 participants • 10 FTD/SQSTM1 carriers • 20 sporadic FTD • 20 healthy controls	European ancestry	ANOVA	• Predominantly right-sided cortical atrophy pattern in FTD patients with SQSTM1 mutations, specifically in fronto-orbito-insular regions and corticospinal projections
(Shinagawa et al. 2015)	Frontotemporal dementia	*C9ORF72* mutation	17 participants • 4 patients with delusions • 13 patients without delusions	Not specified	Two-sample *t*-tests	• C9ORF72 carriers frequently had comorbidities and parietal atrophy
(Yokoyama et al. 2015)	Behavioral variant frontotemporal dementia	5-HTTLPR genotype	203 participants • 24 with bvFTD • 179 controls	Caucasian	Linear regression models	• Patients with bvFTD had smaller volumes in the left inferior frontal gyrus and larger volumes in the right temporal lobe
(Lee et al. 2014)	Behavioral variant frontotemporal dementia	*C9orf72* mutation	42 participants • 14 C9orf72 carriers • 14 non-carriers • 14 healthy controls	Not specified	Two-sample *t*-test	• In C9orf72 carriers, reduced salience network connectivity was linked to atrophy in the left medial pulvinar thalamic nucleus
(Mahoney et al. 2014)	Behavioral variant of frontotemporal dementia	*MAPT, PGRN,* and *C9ORF72* mutations	72 participants • 27 bvFTD • 25 AD patients (disease control) • 20 healthy controls	Not specified	Nonparametric permutation testing	• Widespread WM tract damage was identified in bvFTD patients
(Huang et al. 2016)	Alzheimer’s disease	rs3824968 at SORL1	318 participants	Chinese population from Northern Taiwan	ANCOVA	• People with the SORL1 allele A had smaller GM volumes
(Gomar et al. 2016)	Alzheimer’s disease	BDNF val66 met polymorphisms	397 participants • 222 AD • 175 healthy controls	Not specified	Linear mixed models	• BDNF Met carriers showed thinner cortices in posterior cingulate
(Hibar et al. 2015)	Alzheimer’s disease	rs1345203 and rs1213205 pair	1490 participants • 173 AD • 358 MCI • 206 healthy controls • 753 healthy controls (twins and siblings)	Caucasian	Multiple linear regression	• Significant SNP-SNP interaction explains approximately 2% variance in temporal lobe volume
(Luis et al. 2014)	Alzheimer’s disease	*TREM2* variant p.R47H (rs75932628)	168 participants • 18 TREM2 p.R47H Carriers • 75 non-carriers • 75 healthy controls	European ancestry	Full-factorial two-way ANOVA	• Orbitofrontal cortex and anterior cingulate cortex regions show GM loss in TREM2 p.R47H carriers
(Strenn et al. 2021)	Bipolar disorder	Four *IL1B* polymorphisms (rs1143623, rs1143627, and rs16944, and rs1143634)	242 participants • 188 patients • 54 healthy controls	Not Specified	ANCOVA	• rs16944 and rs1143627 SNPs were linked to larger putamen in the left hemisphere
(Ota et al. 2016)	Bipolar disorder	ANK3 genetic variation (rs10761482)	272 participants • 43 patients • 229 healthy controls	Japanese	ANCOVA	• BD non-T-allele carriers showed smaller reductions in the forceps minor • T-allele carriers demonstrated less age-related brain atrophy in some areas independent of diagnosis cerebellum
(Burciu et al. 2018)	Parkinson's disease	rs356219 SNP in SNCA gene	31 participants • 18 rs356219 carriers • 13 non-carriers	European ancestry	independent t-tests	• The risk group exhibited reduced activation in the contralateral posterior putamen
(Vilas et al. 2016)	Parkinson’s Disease	LRRK2 mutation	36 participants • 18: LRRK2 mutation carriers • 18: non-carriers	European (Spanish) ancestry	GLM	• No appreciable structural changes in asymptomatic LRRK2 mutation carriers when • Compared to healthy controls • LRRK2 mutation carriers had altered functional connectivity in certain brain regions notably decreased connectivity between motor areas and increased connectivity in visual areas
(Dalvie et al. 2017)	Alcohol use disorder	variant (rs219927) in the GRIN2B gene	116 participants • 58 with AUD • 58 healthy control	Mixed ancestry ethnicity	Linear regression model	• The risk group had increased volume in the left posterior cingulate cortex a brain region involved in cognitive function and reward processing
(Dalvie, et al. 2014)	Alcohol use disorder	p.Val66Met allele of BDNF	160 participants • 80 with AUD • 80 healthy control	Mixed ancestry ethnicity	ANCOVA	• No significant associations were found
(Cousijn et al.^2014^)	Schizophrenia	Polymorphisms in MIR137, TCF4, and ZNF804 A genes	1300 participants	Not specified	ANCOVA	• No significant impact of these genes on total brain volume, GM, WM, or hippocampus volume
(Kalmady et al. 2014)	Schizophrenia	rs1800795 SNP in IL- 6 gene	65 participants • 28 Schizophrenia Patients • 37 healthy control	Indian	ANCOVA	• There was an association between the rs1800795 SNP and schizophrenia on hippocampal volume
(Faber et al. 2018)	Hereditary spastic paraplegia	*SPG11* gene mutations	50 participants • 25 patients with SPG11 mutations • 25 healthy control	Not specified	GLM	• Significant differences were found between patients and controls in bilateral motor cortices, limbic structures, and some associative areas
(Rezende et al. 2015)	Hereditary spastic paraplegia	(SPG4-HSP) mutation	34 participants • 11 patients • 23 healthy control	Not specified	GLM and two sample *t*-test	• The spinal cord and corticospinal tracts suffered severe damage, but the cortical mantle was found to be largely intact in patients
(He et al. 2021)	Working memory performance	Summary statistics from GWAS from the UK Biobank and the Human Connectome Project	1141 participants	Diverse population	Linear mixed model	• rs76119478 SNP may control working memory by affecting the left cuneus volume based on the negative correlation
(Martin-Brevet et al. 2018)	Autism spectrum disorder	16p11.2 copy number variants	361 participants • 78 individuals with a 16p11.2 deletion • 71 individuals with a 16p11.2 duplication • 212 control individuals	Not specified	GLM	• Results revealed differences in brain morphometry associated with 16p11.2 CNVs, independent of cognitive, language, social, and psychiatric measures • Regional differences were observed in specific areas, with deletions and duplications affecting distinct brain region
(Saito et al. 2014)	Autistic-like traits	*OXTR* rs2254298 A SNP	135 participants	Japanese	Regression analysis	• Insula volume correlates with autistic-like traits in males • OXTR rs2254298 A allele was linked to smaller insula volume in males
(Brandt et al. 2016)	Hereditary neuropathy with liability to pressure palsies	PMP22 gene deletion of chromosome 17p11.2–12	37 participants • 20 patients • 18 healthy control	Not specified	GLM	• Patients show reduced peripheral vision, prolonged VEP latency, and decreased thickness of the retinal nerve fiber
(Dannlowski et al. 2015)	Major depressive disorder	NCAN gene rs1064395 genotype	683 participants • 171 patients • 512 healthy control	European ancestry	*t*-tests	• A-allele carriers had smaller amygdala and hippocampal volumes, regardless of depression status
(Liu et al. 2015)	Migraine	COMT Val(158) met genotype	246 participants • 135 patients • 111 healthy control	Not specified	two-way ANOVA, and two-sample *t*-test	• Migraineurs with the Val/Val genotype had more GM in the hippocampal region • There was a negative correlation between increased anxiety and Val/Val carriers and decreased functional connectivity between the medial prefrontal cortex and the hippocampal regions

## Results

### Study Selection and Characteristics

**Fig. 2:**
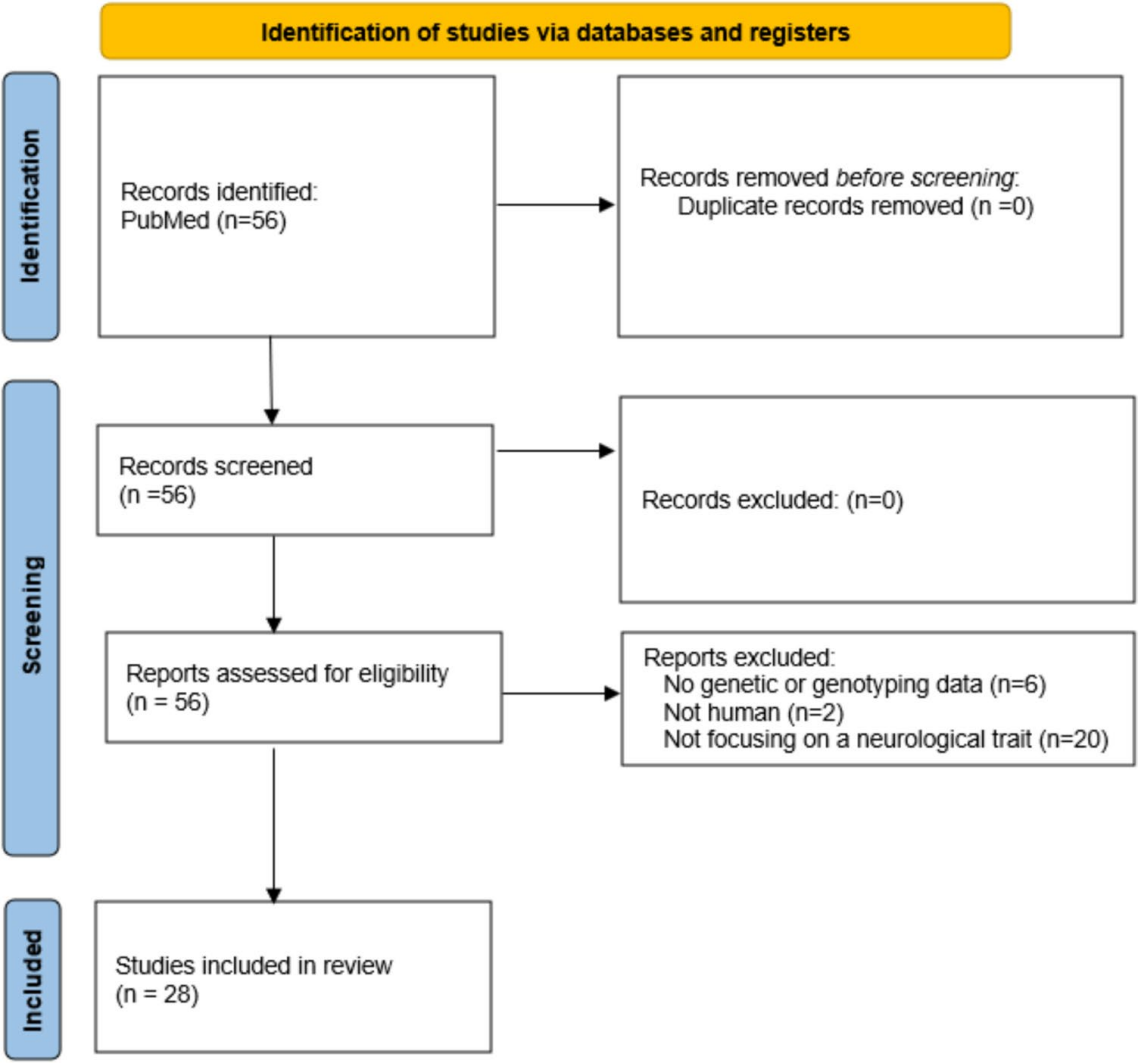
PRISMA flowchart

**Fig. 3:**
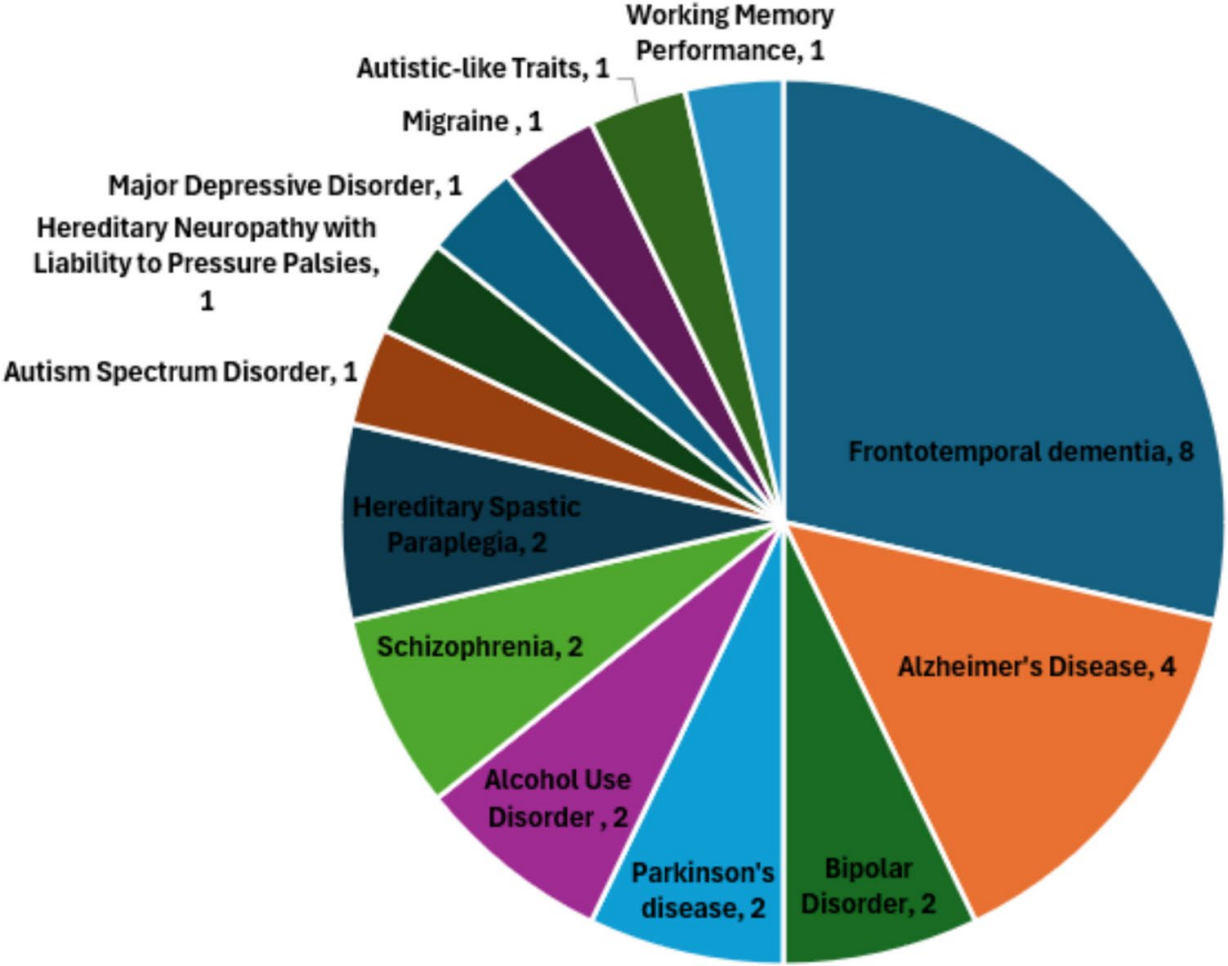
Distribution of neurological phenotypes in the selected studies

A total of 56 articles were retrieved from PubMed, 0 were removed as duplicates, and the 56 full-text articles were screened. Six of them were excluded because no genetic/genotyping data were presented, 2 involved non-human subjects, and 20 did not focus on a neurological trait, leaving 28 studies for final inclusion. Figure 2 provides a PRISMA flowchart detailing the screening process. Out of the included studies, 13 were cross-sectional, 3 were cohort, and 12 were case–control. The distribution of neurological phenotypes in the chosen studies is shown in Fig. 3. The relevant articles that met the inclusion criteria were acquired; then, the relevant data were extracted from the articles. The year of publication, the phenotype under investigation, data used, methodology, and the results for each article were all collected and summarized in Table 1.

### Risk of Bias in Included Studies

**Fig. 4:**
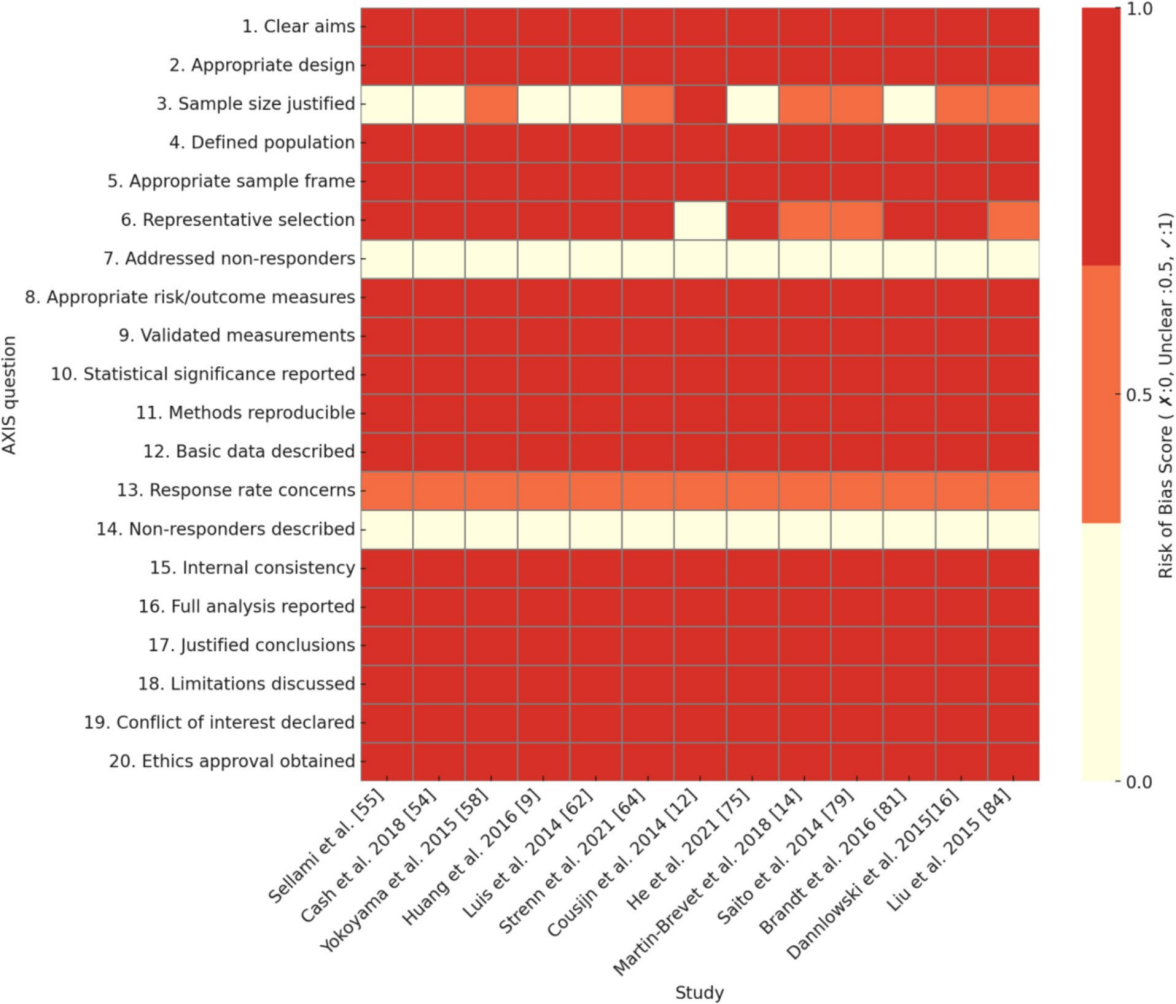
Heatmap of bias reporting across cross-sectional studies (AXIS)

**Fig. 5:**
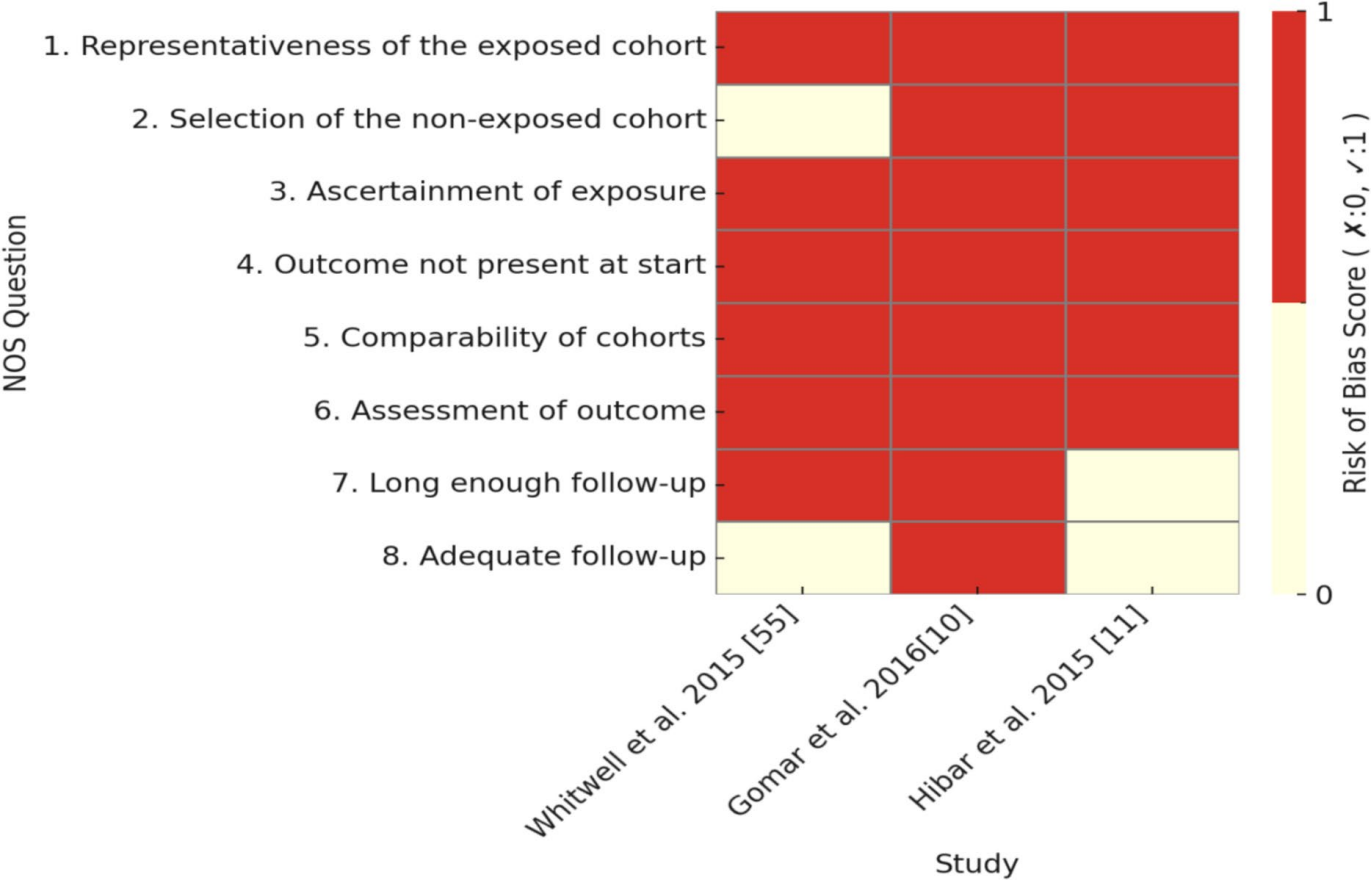
Heatmap of bias assessment using NOS for cohort studies

**Fig. 6:**
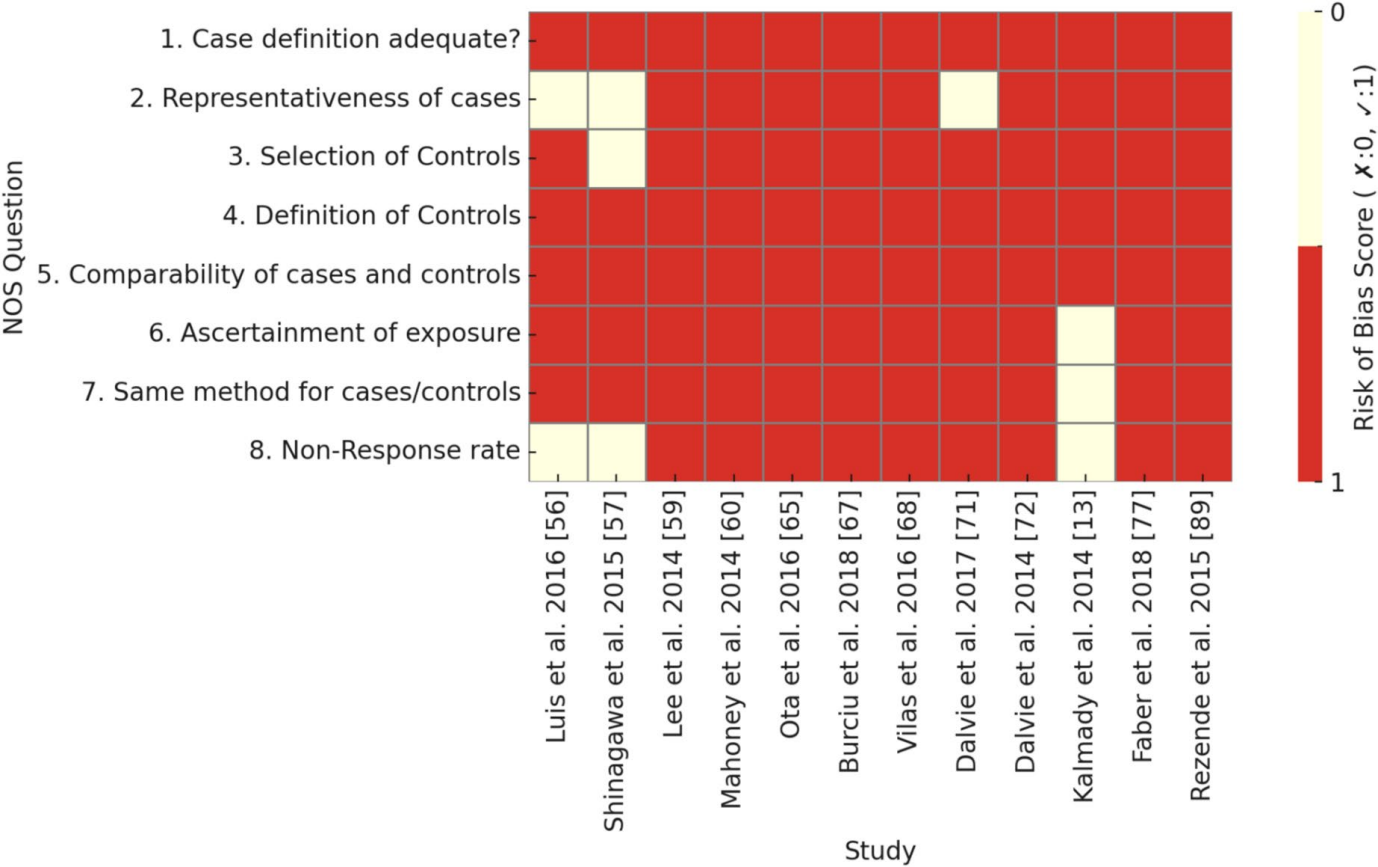
Heatmap of bias assessment using NOS for case–control studies

The risk of bias across the studies in Tables 2, 3, and 4 varies depending on the study design and methodological rigor. Each type of study—cross-sectional, cohort, and case–control—presents specific strengths and limitations that influence the overall risk of bias. Figures 4, 5, and 6 provide heatmaps representing the risk of bias assessment results for cross-sectional, cohort, and case–control studies, respectively. The visualizations highlight commonly underreported or high-risk areas across studies, such as sample size justification, handling of non-responders, and representativeness of the selected population.

**Table 2:** Risk of bias assessment using AXIS for cross-sectional studies

Question	(Whitwell et al. 2015)	(Cash et al. 2018)	(Yokoyama et al. 2015)	(Huang et al. 2016)	(Luis et al. 2014)	(Strenn et al. 2021)	(Cousijn et al. 2014)	(He et al. 2021)			(Martin-Brevet et al. 2018)			(Saito et al. 2014)			(Brandt et al. 2016)			(Dannlowski et al. 2015)					(Liu et al. 2015)	
1. Were the aims/objectives of the study clear?	✓	✓	✓	✓	✓	✓	✓	✓			✓			✓			✓			✓					✓	
2. Was the study design appropriate for the stated aim(s)?	✓	✓	✓	✓	✓	✓	✓	✓			✓			✓			✓			✓					✓	
3. Was the sample size justified?	*x*	*x*	Unclear	*x*	*x*	Unclear	✓	*x*			Unclear			Unclear			*x*			Unclear					Unclear	
4. Was the target/reference population clearly defined?	✓	✓	✓	✓	✓	✓	✓	✓			✓			✓			✓			✓					✓	
5. Was the sample frame taken from an appropriate population base so that it closely represented the target/reference population under investigation?	✓	✓	✓	✓	✓	✓	✓	✓			✓			✓			✓			✓					✓	
6. Was the selection process likely to select subjects/participants that were representative of the target/reference population under investigation?	✓	✓	✓	✓	✓	✓	✓	✓			Unclear			Unclear			✓			✓					Unclear	
7. Were measures undertaken to address and categorize non-responders?	*x*	*x*	*x*	*x*	*x*	*x*	*x*	*x*			*x*			*x*			*x*			*x*					*x*	
8. Were the risk factor and outcome variables measured appropriate to the aims of the study?	✓	✓	✓	✓	✓	✓	✓	✓			✓			✓			✓			✓					✓	
9. Were the risk factor and outcome variables measured correctly using instruments/measurements that had been trialed, piloted or published previously?	✓	✓	✓	✓	✓	✓	✓	✓			✓			✓			✓			✓					✓	
10. Is it clear what was used to determined statistical significance and/or precision estimates? (e.g., *p*-values, confidence intervals)	✓	✓	✓	✓	✓	✓	✓	✓			✓			✓			✓			✓					✓	
11. Were the methods (including statistical methods) sufficiently described to enable them to be repeated?	✓	✓	✓	✓	✓	✓	✓	✓			✓			✓			✓			✓					✓	
12. Were the basic data adequately described?	✓	✓	✓	✓	✓	✓	✓	✓			✓			✓			✓			✓					✓	
13. Does the response rate raise concerns about non-response bias?	Unclear	Unclear	Unclear	Unclear	Unclear	Unclear	Unclear	Unclear			Unclear			Unclear			Unclear			Unclear					Unclear	
14. If appropriate, was information about non-responders described?	*x*	*x*	*x*	*x*	*x*	*x*	*x*	*x*			*x*			*x*			*x*			*x*					*x*	
15. Were the results internally consistent?	✓	✓	✓	✓	✓	✓	✓	✓			✓			✓			✓			✓					✓	
16. Were the results presented for all the analyses described in the methods?	✓	✓	✓	✓	✓	✓	✓	✓			✓			✓			✓			✓					✓	
17. Were the authors’ discussions and conclusions justified by the results?	✓	✓	✓	✓	✓	✓	✓	✓			✓			✓			✓			✓					✓	
18. Were the limitations of the study discussed?	✓	✓	✓	✓	✓	✓	✓	✓			✓			✓			✓			✓					✓	
19. Were there any funding sources or conflicts of interest that may affect the authors’ interpretation of the results?	✓	✓	✓	✓	✓	✓	✓	✓			✓			✓			✓			✓					✓	
20. Was ethical approval or consent of participants attained?	✓	✓	✓	✓	✓	✓	✓	✓			✓			✓			✓			✓					✓

**Table 3:** Risk of bias assessment using NOS for cohort studies

	Question	(Whitwell et al. May 2015)	(Gomar et al. Mar. 2016)	(Hibar et al. Jan. 2015)
Selection	1. Representativeness of the exposed cohort	★	★	★
	3. Selection of the non-exposed cohort	-	★	★
	5. Ascertainment of exposure	★	★	★
	7. Demonstration that outcome of interest was not present at start of study	★	★	★
Comparability	1. Comparability of cohorts on the basis of the design or analysis	★	★	★
Outcome	1. Assessment of outcome	★	★	★
	2. Was follow-up long enough for outcomes to occur	★	★	-
	3. Adequacy of follow up of cohorts	-	★	-

**Table 4:** Risk of bias assessment using NOS for case–control studies

	Question	Luis et al. 2016)	(Shinagawa et al. 2015)	(Lee et al. 2014)	(Mahoney et al. 2014)	(Ota et al. 2016)	(Burciu et al. 2018)	(Vilas et al. 2016)	(Dalvie et al. 2017)	(Dalvie, et al. 2014)	(Kalmady et al. 2014)	(Faber et al. 2018)	(Rezende et al. 2015)
Selection	1. Is the case definition adequate?	★	★	★	** ★**	** ★**	** ★**	** ★**	** ★**	** ★**	** ★**	** ★**	** ★**
	2. Representativeness of the cases	-	-	★	★	★	★	★	-	★	★	★	★
	3. Selection of controls	★	-	★	★	★	★	★	★	★	★	★	★
	4. Definition of controls	★	★	★	★	★	★	★	★	★	★	★	★
Comparability	1. Comparability of cases and controls on the basis of the design or analysis	★	★	★	★	★	★	★	★	★	★	★	★
Exposure	1. Ascertainment of exposure	★	★	★	★	★	★	★	★	★	-	★	★
	2. Same method of ascertainment for cases and controls	★	★	★	★	★	★	★	★	★	-	★	★
	3. Non-response rate	-	-	★	★	★	★	★	★	★	-	★	★

In Table 2, which includes cross-sectional studies assessed using the AXIS tool, most studies demonstrated clear aims and appropriate study designs. The target and reference populations were well defined, and the measurement tools used to assess risk factors and outcomes were validated in prior research. Statistical significance methods and internal consistency were also well addressed. However, several key limitations increased the risk of bias. Many studies lacked justification for their sample sizes, making it unclear whether they had sufficient power to detect meaningful associations. Additionally, the selection process for participants was not always well described, raising concerns about representativeness. A particularly notable issue was the failure to address non-response bias—none of the studies took measures to categorize non-responders, which could introduce selection bias. Furthermore, some studies did not clearly disclose funding sources or conflicts of interest, leaving open the possibility of external influence on their findings. Due to these limitations, the overall risk of bias in cross-sectional studies is considered moderate.

The cohort studies in Table 3, evaluated using the NOS, generally exhibited a more robust methodological approach, leading to a moderate to low risk of bias. Most studies clearly defined their exposed cohorts, measured risk factors and outcomes appropriately, and maintained comparability between study groups. However, one recurring limitation was the unclear follow-up duration, which raises concerns about whether the studies adequately captured long-term effects. Additionally, some studies did not ensure adequate follow-up of participants, which could lead to attrition bias if a significant number of subjects were lost to follow-up. Another concern was how the non-exposed cohorts were selected—if this were not done properly, selection bias could be introduced.

In Table 4, case–control studies were also assessed using the NOS tool. Most studies adequately defined cases and measured exposures in a reliable manner. Furthermore, many maintained comparability between cases and controls, strengthening the validity of their findings. However, some methodological concerns were observed. The representativeness of cases was not always well documented, and the selection of controls was sometimes unclear, introducing potential selection bias. Additionally, non-response rates were rarely addressed, making it difficult to determine whether systematic differences existed between respondents and non-respondents. Some studies also did not clearly define the criteria for selecting controls, which could further impact the reliability of comparisons.

### Overall Outcomes

Out of the included articles, a variety of neuroimaging and genetic methodologies were used to explore how genetic variations intersect with brain structure across multiple neurological disorders. The most commonly used neuroimaging method was VBM. Several studies also employed GLM, ANCOVA, and other statistical techniques to investigate associations between genetic variants and imaging metrics. One study utilized machine learning algorithms (e.g., EPISIS) to uncover complex interactions among SNPs (Hibar et al. Jan. 2015). The risk of bias varied across studies, with some demonstrating strong methodological rigor, while others had concerns regarding sample size justification and participant selection. The included studies ranged from small sample sizes (~ 30 participants) to larger GWAS studies with over 1000 individuals, impacting the statistical power of findings. Additionally, the heterogeneity in imaging techniques and genetic analysis methods limited direct comparisons across studies. Overall, the quality of evidence for genetic influences on neurological conditions was graded as moderate, suggesting that while there is substantial support for genetic contributions to brain structure and function, methodological limitations remain.

### Disease-Specific Findings

Table 1 provides a summary of the study findings. Across the included studies, multiple neurological disorders were analyzed, with frontotemporal dementia (FTD) appearing in 28.5% of the studies and AD in 15%, and other conditions, such as schizophrenia, bipolar disorder (BD), PD, autism spectrum disorder, major depressive disorder, and hereditary spastic paraplegia, also represented.

#### Frontotemporal Dementia

FTD is broadly divided into semantic dementia (SD), progressive nonfluent aphasia (PNFA), and bvFTD. Sellami et al. (Sellami, et al. 2018) aimed to determine whether distinct neuropsychiatric symptoms (NPS) were associated with genetic mutations related to FTD, specifically in carriers of *MAPT*, *GRN*, or *C9orf72* gene mutations. The study comprised 167 mutation carriers (60 C9orf72, 32 MAPT, and 75 GRN), and the neuroanatomical correlations of NPS were discovered using VBM and a multiple regression model. They discovered that, in those with the C9orf72 mutation, delusions were mainly linked to left frontal cortical atrophy, while cerebellar atrophy was exclusively associated with anxiety and neuropsychiatric symptoms in the MAPT group. Finally, anxiety in the GRN group was linked to GM reduction in the default-mode network’s posterior structures. Cash et al. (Cash et al. Feb. 2018) conducted research on the patterns of GM atrophy with respect to the same three FTD mutations as in Sellami, et al. (2018) using VBM and GLMs for statistical analysis on three groups: 47 mutation carriers who were clinically afflicted, 128 presymptomatic mutation carriers, and 144 non-mutation carriers. The presymptomatic *C9orf72* group showed GM reduction in the thalamus and cerebellum, the MAPT group showed GM reduction in the anterior and medial temporal lobes, and finally, the GRN group showed GM reduction in the striatum, posterior frontal, and parietal lobes. Whitwell et al. (Whitwell et al. May 2015) also investigated whether longitudinal MRI measurements could be used as biomarkers for the previously discussed three genetic forms of FTD studied in Sellami, et al. (2018) and (Cash et al. Feb. 2018), in addition to sporadic FTD. Longitudinal models were fitted over time from the year 1993 to 2012 using linear mixed effects models with random intercepts and slopes that were specific to each subject. They applied Voxel-level comparisons using* t*-tests. They found that the GRN group had the fastest whole-brain atrophy rates and the highest regional atrophy across all lobes. While sporadic FTD demonstrated more atrophy in the anterior cingulate than both *C9ORF72* and *MAPT*, *C9ORF72* demonstrated more reduction in the left cerebellum and right occipital lobe than did *MAPT*. Luis et al. (Luis et al. Jun. 2016) examined the brain atrophy patterns linked to frontotemporal lobar degeneration (FTLD) caused by mutations in the *SQSTM1* gene, using VBM and ANOVA for group comparisons. By acquiring brain MRI scans of FTD patients with SQSTM1 mutations, FTD patients without the mutation, and healthy controls, the results showed that SQSTM1 mutation carriers primarily showed a right-sided pattern of cortical atrophy, particularly in fronto-orbito-insular regions and corticospinal projections. Shinagawa et al. (2015) investigated the relationship between FTLD delusions and *C9ORF72* mutations. Four of the 17 patients with FTLD and *C9ORF72* mutations showed signs of delusions. The MRI for the two groups was compared using group comparison of VBM. The study found that delusions in C9ORF72 carriers frequently accompany comorbidities and parietal atrophy. Yokoyama et al. (Yokoyama et al. 2015) investigated how the 5-HTTLPR short allele, which is linked to anxiety and depression, affected the GM in both bvFTD patients and healthy controls using VBM and linear regression models. The findings demonstrated that patients with bvFTD had smaller volumes in the left inferior frontal gyrus and larger volumes in the right temporal lobe, respectively, when they carried more copies of the 5-HTTLPR allele. Lee et al. (Lee et al. Nov. 2014) investigated brain network integrity in patients with bvFTD, both with and without the *C9orf72* mutation, by comparing smooth GM maps using VBM and two-sample *t*-tests. Although the two groups displayed different patterns of brain atrophy, they both had decreased connectivity in the salience and sensorimotor networks. Network connectivity in the default mode was higher among carriers who were not *C9orf72.* A decrease in the salience network connectivity linked to atrophy in the left medial pulvinar thalamic nucleus in C9orf72 carriers. Functional MRI had demonstrated potential in identifying C9orf72 carriers at early stages of the disease and could act as a common biomarker for all anatomical variations. Finally, Mahoney et al. (Mahoney et al. Aug. 2014) examined white matter (WM) changes in patients with bvFTD using DTI. Using VBM and nonparametric permutation testing, they investigated GM volume in different regions between groups. When bvFTD patients were compared to healthy controls and AD patients, extensive WM damage was discovered, with the uncinate fasciculus, cingulum bundle, and corpus callosum showing prominent involvement. Beyond regions of GM atrophy, WM changes were observed in patients, indicating a distinct role for WM disruption in the pathophysiology of the disease that may be impacted by underlying molecular pathologies.

#### Alzheimer’s Disease

AD appeared in 15% of the selected studies. AD is a progressive neurodegenerative disease affecting families in cognition and behavior functions. Over 100 million people worldwide are expected to be impacted by AD by the year 2050 (Ulep et al. 2018). Huang et al. (Huang et al. 2016) examined the relationship between the GM volume in healthy Chinese adults and AD patients with SORL1 rs3824968 as the genetic variant. Using VBM and ANCOVA, they found that people with the SORL1 allele A had smaller GM volumes in particular brain regions than people without the allele, implying that SORL1 rs3824968 affects brain structure across the lifespan and raises the risk of AD, in addition to possibly hastening age-related brain changes. Gomar et al. (Gomar et al. 2016) investigated the effect of BDNF val66 met polymorphisms on the temporal lobe structure and memory using mixed linear models. Thinner posterior cingulate and precuneus cortices were found in healthy APOE E4 gene carriers who carried the allele. Furthermore, the allele was associated with a quicker decline in entorhinal cortex thickness and worse memory function in people with mild cognitive impairment or AD who carry the APOE E4 gene. This shows that having the BDNF Met allele may prevent compensatory brain mechanisms in people with the APOE E4 gene, resulting in accelerated age-related brain changes and cognitive decline. Hibar et al. (Hibar et al. 2015) used EPISIS, a machine learning algorithm, to identify SNP-SNP genetic interactions affecting brain volume in AD. Using tensor-based morphometry (TPM) and multiple linear regression, they found a significant interaction between two SNPs (*rs1345203* and *rs1213205*) that explained the variability of approximately 2% of temporal lobe volume. This interaction was associated with greater brain volume, suggesting a protective effect against the disease. The clinical neuropsychological and neuroimaging characteristics connected to the uncommon TREM2 p.R47H variant in AD were examined by Luis et al. (Luis et al. 2014) using AD and mild cognitive impairment (MCI) patients from two sample sets (Spanish and ADNI) having the R47H variant. The orbitofrontal cortex and anterior cingulate cortex in both datasets showed significant GM loss in brain imaging analysis using VBM and full-factorial ANOVA, indicating that these regions are particularly vulnerable to the TREM2 p. R47H variant. The study concludes that this variant, which primarily affects frontobasal and temporal regions, is associated with particular clinical and neuroimaging features in AD.

#### Bipolar Disorder

BD appeared in two of the selected studies. BD is a chronic disease that recurs frequently and is characterized by mood and energy swings. It impacts over 1% of the population worldwide, regardless of socioeconomic status, nationality, or ethnic background. It is an important factor of disability in young people that may end with suicide death (Grande et al. 2016). The relationship between SNPs in the IL1B gene and the volume of brain GM in BD patients was examined by Strenn et al. (Strenn et al. 2021). VBM and ANCOVA were used to examine the impact of the SNPs on GM volume. Thus, in both BD patients and controls, they discovered that *rs16944* and *rs1143627* SNPs were linked to larger putamen in the left hemisphere. ANK3 gene variation (rs10761482) effects on brain structure in BD patients and healthy controls were studied by Ota et al. (Ota et al. 2016) using VBM and ANCOVA. In comparison to controls, BD patients’ brain regions exhibited reduced fractional anisotropy based on VBM and MRI data. Significantly, BD non-T-allele carriers showed smaller reductions in the forceps minor. Furthermore, T-allele carriers demonstrated less age-related brain atrophy in some areas, independent of diagnosis. These results imply that, in both healthy individuals and BD patients, the ANK3 gene may have an impact on age-related changes in brain structure.

#### Parkinson’s Disease

PD appeared in two of the selected studies. With a variety of etiologies and clinical manifestations, PD is a recognized clinical syndrome that is a rapidly developing neurodegenerative disorder whose prevalence is rising globally (Bloem et al. 2021). Burciu et al. (Burciu et al. 2018) investigated healthy old participants with the *rs356219* SNP, which is previously identified as a risk factor for developing PD. The study used multimodal MRI and various motor and cognitive tests. VBM and independent *t*-tests were used to discover the change in different brain regions functional connectivity, iron content in the substantia nigra, and group differences in GM density and cortical thickness. Task-based fMRI showed less activation in the contralateral posterior putamen in the risk group. Vilas et al. (Vilas et al. 2016) used VBM and GLMs to better understand the prediagnostic phase of LRRK2-associated PD by characterizing resting-state functional connectivity patterns in carriers of the mutation in asymptomatic individuals using MRI. There were no appreciable structural changes when compared to healthy controls in asymptomatic LRRK2 mutation carriers, but they did exhibit altered functional connectivity in certain brain regions, notably decreased connectivity between motor areas and increased connectivity in visual areas.

#### Alcohol Use Disorder

Alcohol use disorder (AUD) appeared in two of the selected studies. AUD disorders are characterized by obsessive heavy alcohol consumption and a loss of control over alcohol intake (Carvalho et al. Aug. 2019). It is a condition of different brain circuits connected to cognitive functions that results in an excessive intake of alcohol despite negative effects on one’s health and social life (Yang et al. 2022). Dalvie et al. (Dalvie et al. 2017) investigated the relationship between brain volume and genetic variations associated with psychiatric disorders in teenagers with AUD. A specific variant (*rs219927*) in the *GRIN2B* gene was linked with increased volume in the left posterior cingulate cortex, a brain region involved in cognitive functions and reward processing based on genotyping and magnetic resonance imaging data of 58 adolescents with AUD and 58 matched controls using VBM and linear regression modeling. Dalvie et al. (Dalvie, et al. 2014) investigated the BDNF p.Val66Met polymorphism, AUD, and early life adversity on brain volume in adolescents with childhood trauma using 2 × 2 ANCOVA analysis using VBM. Additionally, the interaction between genotype and childhood trauma questionnaire score was examined to assess potential combined effects on brain volume alterations. No significant associations were found between these factors after correcting for multiple comparisons.

#### Schizophrenia

Schizophrenia appeared in two of the selected studies. Psychosis and a reduction in functioning are symptoms of schizophrenia, a long-term disorder. Neurotransmitters, particularly dopamine, interact in the frontal, temporal, and mesostriatal brain regions, causing schizophrenia symptoms (Kesby et al. 2018). Despite years of research, many factors related to the intricate pathophysiology of schizophrenia are still not identified. According to a set of genetic studies, schizophrenia is highly pleiotropic and involves multiple genetic loci (Owen et al. 2016). Cousijn et al. (Cousijn et al. 2014) examined the potential effects of polymorphisms in the MIR137, TCF4, and ZNF804 A genes on adult brain structure. Using full-factorial ANCOVA, the researchers found no significant impact of these genes on total brain volume, GM, WM, or hippocampus volume. This suggests that these genes influence on brain morphometry is probably not the reason for the association between them and schizophrenia. The relationship between the rs1800795 SNP in the *IL- 6* gene and hippocampal GM volume in patients with antipsychotic-naïve schizophrenia and healthy controls was examined by Kalmady et al. (Kalmady et al. May 2014). Patients showed significantly smaller bilateral hippocampus volumes. There was an association between the rs1800795 SNP and schizophrenia on hippocampal volume when applying VBM and ANCOVA. This implies that the SNP in the *IL- 6* gene might have a sex-specific impact.

#### Other Disorders

Working memory is a fundamental cognitive function of humans that allows them to temporarily store and manipulate information while carrying out complex tasks, such as learning, reasoning, and comprehension. He et al. (He et al. 2021) investigated the relationship between genetic variations, brain morphometry, and WM performance. They used imaging data and summary statistics from GWAS from the UK Biobank and over 1114 participants in the Human Connectome Project (HCP). Linear mixed model (LMM)–based mediation analysis was conducted to investigate possible mediated relationships between imaging morphometric phenotypes and working memory performance in the HCP dataset. It was determined that the rs76119478 SNP may control working memory by affecting the left cuneus volume based on the negative correlation.

With a prevalence of 0–9 times per 100,000 people worldwide, hereditary spastic paraplegias (HSPs) are a diverse group of monogenic neurological defects that contribute to corticospinal and dorsal spinal cord axonal atrophy. The main clinical symptom of this uncommon neurodegenerative illness is spasticity in the lower extremities (Meyyazhagan and Orlacchio 2022). Faber et al. (Faber et al. 2018) investigated the anatomical abnormalities, clinical measures, and neuronal susceptibility in HSP patients with SPG11 gene mutations. The study examined patients with confirmed SPG11 mutations from 16 unrelated families, comparing their neurological, neurophysiological, and neuroimaging data to a control group. VBM and GLMs were utilized for group comparisons between patients and controls. They found that cortical thickness analysis revealed significant differences between patients and controls in bilateral motor cortices, limbic structures, and some associative areas, while WM tracts also showed significant alterations in patients compared to controls. These findings suggest widespread structural brain abnormalities in patients. Also, Rezende et al. (He et al. 2021) looked into the degree of neurodegeneration in individuals who had SPG4 gene mutation-related HSPs using VBM. The tractography results of the two groups were compared, and differences in fractional anisotropy, mean diffusivity, radial diffusivity, and axial diffusivity were examined using a two-sample *t*-test. A GLM was utilized to evaluate cortical thickness variation between the two groups. The spinal cord and corticospinal tracts suffered severe damage, but the cortical mantle was found to be largely intact. It appears that these regions are crucial to the development of hereditary spastic paraplegia because the damage was correlated with the severity of the disease.

The neurodevelopmental disorder known as autism spectrum disorder (ASD), or autism, is a prevalent, highly heritable, and heterogeneous disorder with underlying cognitive features that frequently co-occur with other conditions. ASD is used to characterize people who exhibit a particular set of difficulties with social communication, repetitive behaviors, and severely limited interests and/or sensory behaviors that start early in life (Lord et al. 2020).

Martin-Brevet et al. (Martin-Brevet et al. 2018) investigated the effects of 16p11.2 copy number variants (CNVs) on brain GM volume using MRI data from individuals with deletions, duplications, and controls, using VBM and GLMs. Results revealed differences in brain morphometry associated with 16p11.2 CNVs, independent of cognitive, language, social, and psychiatric measures. While global effects showed a mirror pattern (deletion > control > duplication), regional differences were observed in specific areas, with deletions and duplications affecting distinct brain regions.

Autism-like traits (ALTs) are observed as difficulties or abnormalities in sociocommunicative behavior, self-perception, and environmental adaptations. They can be thought of as a phenotype arising from the overlap of genetic factors for ASDs (Saito et al. 2014). (Saito et al. 2014) investigated the connection between brain anatomy and ALTs, specifically as it relates to the oxytocin receptor gene (OXTR) using VBM and regression analysis. Researchers found that lower prosociality (a component of ALTs) was connected with smaller GM volume in the right insula in males. They found that the OXTR rs2254298 A allele, a genetic variant linked to an increased risk of autism, was connected to this decrease in insula volume.

Hereditary neuropathy with liability to pressure palsies (HNPP) is distinguished by focal conduction abnormalities at entrapment sites on nerve conduction studies, sausage-like swellings (tomacula) of the myelin sheaths by nerve biopsy, and recurrent sensory and motor neuropathy in individual nerves beginning in adolescence or young adulthood (Attarian et al. 2020). Brandt et al. (Brandt et al. 2016) used a linear model for VBM to examine local GM volume variations between HNPP patients with PMP22 gene deletion and healthy controls. Patients showed less peripheral vision and displayed a longer visual evoked potential (VEP) latency. Prolonged VEP latency was found to be correlated with decreased thickness of the retinal nerve fiber layer in the nasal region in the patients. Furthermore, magnetic resonance spectroscopy showed that the patients’ visual cortex had fewer metabolites. Nonetheless, there was no significant difference observed between the groups in terms of whole brain volume, GM, WM volume, and metabolites in a sensory cortex control voxel.

Around 185 million people worldwide suffer from major depressive disorder (MDD), a common mental disorder. Depressive mood, diminished interest in or enjoyment from previously enjoyable activities, and recurrent thoughts of death are some of the symptoms of MDD. A variety of factors, including genetics, environment, biology, and psychology, appear to be combined to make MDD a complex disorder (Marx et al. 2023). Dannlowski et al. (Dannlowski et al. 2015) investigated the association between the NCAN rs1064395 gene variant, previously linked to mental disorders, and GM volume in the brain using VBM and *t*-tests. They hypothesized that the risk (A) allele of this variant would be connected with reduced GM volumes in emotion-processing regions like the amygdala and hippocampus. They found that A-allele carriers had smaller amygdala and hippocampal volumes, regardless of depression status. These findings suggest that the NCAN genotype may contribute to structural brain differences in areas crucial for emotion regulation, potentially increasing vulnerability to mental disorders in the presence of additional risk factors.

According to global statistics, migraine is the second most common neurological condition that causes years of disability (Ashina et al. 2021). (Liu et al. 2015) investigated hippocampal structure and function when migraine and the COMT Val(158)met genotype were combined. To evaluate the impact of disease state genetic variations and their combination on brain morphometry, a two-way ANOVA was utilized. Furthermore, a seed-based method was used to perform functional connectivity (FC) analysis, with the hippocampus serving as the seed region. The study employed a two-sample *t*-test to assess the differences in FC between the groups. When compared to healthy controls who had the same genotype, they discovered that migraineurs with the Val/Val genotype had more GM in the hippocampal region. Furthermore, there was a negative correlation between increased anxiety and Val/Val carriers and decreased functional connectivity between the medial prefrontal cortex and the hippocampal regions.

Finally, studies employed diverse imaging modalities (structural MRI with voxel-based morphometry, diffusion tensor imaging, fMRI) and a variety of genetic analysis methods (candidate gene association, GWAS, SNP-SNP interaction). This heterogeneity complicates direct cross-study comparisons while highlighting the broad application of imaging genetics in addressing complex neurological disorders.

## Discussion

The reviewed studies collectively demonstrate the growing importance of imaging genetics in advancing our understanding of neurological and psychiatric disorders. Among the methodologies employed, voxel-based morphometry VBM was the most frequently used neuroimaging technique, due to its sensitivity in detecting subtle GM volume changes. This makes VBM particularly valuable for disorders such as AD, FTD, and schizophrenia, where neurodegeneration is a central feature. In contrast, studies employing DTI and fMRI (Lee et al. 2014) provided stronger evidence linking structural and functional alterations to specific genetic variants, particularly in PF and FTD, where connectivity disruptions often occur before visible atrophy. However, the limited application of machine learning-based methods, such as EPISIS, has constrained the discovery of non-linear genetic interactions, likely due to computational demands and limited adoption in imaging genetics studies. EPISIS, which models SNP-SNP interactions in imaging genetics, was applied in only one study (Hibar et al. 2015), limiting its potential for uncovering complex genetic influences on brain structure and function. Expanding AI-driven methods in imaging genetics could enhance the detection of complex genotype–phenotype relationships. Another limitation is the heterogeneity in statistical methodologies. While GLMs and ANCOVA were frequently used, many studies suffered from small sample sizes, reducing statistical power. Large-scale consortium efforts, such as ENIGMA and UK Biobank, are crucial for improving reproducibility and generalizability.

Despite the frequent use of statistical significance testing, many studies in this review did not report effect sizes, confidence intervals (Cis), or measures of practical significance. This limits interpretability, especially when statistical significance may result from large sample sizes or multiple comparisons. While the lack of CIs limits the interpretability of precision and the reliability of the reported estimates, only a few studies reported confidence intervals to indicate the precision of their estimates (Gomar et al. 2016; He et al. 2021). Most studies did not report any standardized effect sizes, such as Cohen’s *d* or Hedges’ *g*. Only a few exceptions (Gomar et al. 2016; Martin-Brevet et al. 2018; Burciu et al. 2018; He et al. 2021) included effect sizes, which added valuable information about the magnitude of findings. Gomar et al. (Gomar et al. 2016) and Burciu et al. (Burciu et al. 2018) notably used Hedges’ g, appropriate for small samples, while He et al. (He et al. 2021) and (Martin-Brevet et al. 2018) used Cohen’s *d*, which is one of the most widely used metrics in neuroimaging and clinical research to express standardized mean differences. The absence of standardized reporting practices, including the omission of essential statistical metrics like effect size and confidence intervals, complicates cross-study comparisons and reduces the transparency and reproducibility of findings. Future imaging genetics research should follow established reporting standards by including both statistical significance and practical effect measures, ensuring a more comprehensive understanding of genotype–phenotype relationships.

Multimodal data integration, which combines different imaging techniques or integrates imaging with genetic analyses, enhances the understanding of genotype–phenotype relationships by capturing network-level disruptions that single-modal approaches often miss. Traditional single-modal studies provided valuable insights into brain structure and function but lacked direct links to genetic factors. For example, VBM and fMRI studies identified abnormalities in AD (Li et al. 2020; Chelladurai et al. 2023) and schizophrenia (Algumaei et al. 2022; Juneja et al. 2018), yet could not determine whether these were driven by genetic risk factors. Similarly, genetic studies using GWAS or candidate genes often lacked phenotypic validation, limiting their ability to assess genetic influences on brain morphology or connectivity. In contrast, multimodal imaging genetics integrates both neuroimaging and genetic data, offering a more comprehensive perspective on genotype–phenotype relationships. In contrast to single-modal approaches that focus only on localized structural changes (e.g., VBM for gray matter atrophy or GWAS for genetic associations), multimodal methods enable the identification of broader network-level disruptions and functional alterations. For instance, integrating functional MRI with genetic data can identify disruptions in brain connectivity patterns linked to specific genetic risk factors, as seen in studies on PD and FTD. This approach has been particularly useful in neurodegenerative disorders, where structural abnormalities alone do not fully capture disease mechanisms. However, while multimodal approaches enhance disease classification and early diagnosis, challenges such as data complexity, standardization, and computational demands must be addressed to improve reproducibility and cross-study comparisons. Studies incorporating DTI and fMRI (Mahoney et al. 2014; Lee et al. 2014) with genetic analysis, for example, have been more successful in linking structural and functional alterations to specific genetic variants, as seen in studies of PD and FTD. However, while multimodal studies provide richer insights, they also introduce challenges related to sample size requirements and computational complexity, limiting their widespread adoption compared to single-modal approaches.

One common feature is the overlapping genetic contributions across multiple neurological conditions. The APOE and TREM2 variants, associated with AD, were found to affect GM loss in the posterior cingulate, orbitofrontal cortex, and anterior cingulate cortex (Gomar et al. 2016). Among the most studied genetic variants, the COMT Val158Met polymorphism was frequently associated with prefrontal cortex and hippocampal function, particularly in schizophrenia and mood disorders, where it influenced cognitive control and emotional regulation (Liu et al. 2015). Second, genes that influence synaptic plasticity and neural connectivity (e.g., BDNF, COMT) appear in both degenerative and psychiatric conditions, implying convergent molecular pathways influencing brain structure. This supports the hypothesis that psychiatric and neurodegenerative disorders share underlying genetic mechanisms, an area that needs further investigation. FTD-related studies demonstrated distinct patterns of neurodegeneration based on genetic mutations (C9orf72, MAPT, GRN), suggesting that genotype-specific atrophy patterns could inform early diagnostic biomarkers.(Sellami, et al. 2018) (Cash et al. 2018) (Shinagawa et al. 2015) (Lee et al. 2014). Studies on BDNF polymorphisms revealed associations with hippocampal volume loss in depression and anxiety disorders (Gomar et al. 2016) (Dalvie, et al. 2014). Additionally, altered connectivity patterns linked to SNCA and LRRK2 variants in PD were detected in mutation carriers before the onset of motor symptoms, reinforcing the potential of imaging genetics in early disease detection (Burciu et al. 2018). Genetic variations in DISC1 were found to modulate prefrontal-limbic connectivity in schizophrenia, and hereditary spastic paraplegia was strongly associated with mutations in SPG4 and SPG11, leading to progressive degeneration of corticospinal tracts (He et al. 2021) (Faber et al. 2018) (Rezende et al. 2015).

Imaging genetics research faces critical challenges. A major challenge is the small sample sizes, which reduce statistical power, limit the detection of genotype–phenotype associations, increase the risk of false positives or non-replicable findings, and limit generalizability. Future studies should prioritize multicenter collaborations and meta-analyses to enhance sample sizes and ensure findings are robust across different populations. Another significant limitation is the lack of multimodal imaging integration in most studies. The widespread use of VBM emphasizes structural alterations but does not capture the functional and network-level changes that often precede visible atrophy. DTI and fMRI were used in only a few studies, despite their potential to detect early disease biomarkers and connectivity disruptions in disorders like PD and FTD. Integrating different imaging techniques will provide a more comprehensive view of the genetic influence on brain structure and function, improving both diagnostics and treatment strategies. A further challenge lies in the heterogeneity of data collection, preprocessing methods, and statistical analyses across studies. Differences in genotyping platforms, imaging protocols, and preprocessing pipelines complicate direct cross-study comparisons, limiting reproducibility. Establishing common frameworks for data acquisition, processing, and analysis across research institutions—such as those developed by large-scale initiatives like ENIGMA—will be essential for ensuring reproducibility and comparability of results. Also, the application of machine learning and artificial intelligence in imaging genetics remains limited despite its potential to discover complex, non-linear relationships between genetic variations and brain phenotypes. While some studies employed AI-driven approaches, such as EPISIS in AD (Hibar et al. 2015), their adoption is still in its early stages. Machine learning methods must be carefully implemented to prevent overfitting and enhance interpretability, particularly in complex genetic and neuroimaging analyses. Future research should prioritize explainable AI models and deep learning frameworks that can extract meaningful genetic-imaging associations while ensuring transparency in computational decision-making. Finally, the translation of imaging genetics findings into clinical practice remains a major concern. Although certain genetic markers, such as APOE genotyping in AD, are already being used in risk assessment, most imaging-genetics associations have yet to be validated for routine medical use. A key obstacle is the complexity of polygenic disorders, where genetic influences on brain structure are often indirect and mediated by multiple interacting variants and environmental factors. Future efforts should focus on integrating imaging genetics with multi-omics data and longitudinal studies to improve disease prediction, patient stratification, and personalized treatment approaches. By overcoming these barriers, imaging genetics has the potential to revolutionize the diagnosis and treatment of neurological disorders, laying the foundation for precision medicine. Despite these challenges, integrating genetic risk measures with imaging biomarkers represents a promising step toward actionable precision diagnostics.

Translating imaging genetics findings into clinical practice remains a significant challenge. The polygenic nature of neurological disorders adds complexity, as genetic influences are often indirect and shaped by interactions among multiple variants and environmental factors. Polygenic risk scores (PRS), which aggregate the effects of multiple genetic variants associated with a disorder, offer a promising path for risk stratification in clinical settings. When combined with neuroimaging-derived biomarkers, such as structural atrophy patterns or functional connectivity disruptions, these multimodal signatures can improve the early identification of at-risk individuals, even before symptom onset. For instance, in AD and PD, PRS could be used to select high-risk individuals for more frequent monitoring or inclusion in prevention trials (Baker and Escott-Price 2020; Jacobs et al. 2020). In clinical settings, these multimodal risk signatures could be implemented via electronic health records, integrating genomic screening with routine MRI to identify high-risk individuals for early monitoring or preventive trials. Additionally, imaging genetic profiles could help distinguish between overlapping symptom clusters and inform tailored intervention strategies, particularly in the early stages of disorders like schizophrenia or BD (Jiang et al. 2019; Chen et al. 2019). To support clinical integration, future studies should focus on validating these biomarkers in large, diverse clinical populations, ensuring reproducibility and developing decision support tools for clinicians.

Despite the promise of imaging genetics, this review is constrained by several limitations. First, the relatively small sample sizes in certain studies may limit the statistical power to detect gene-brain associations. Second, variability in imaging protocols and genetic analyses—such as different preprocessing pipelines or genotyping platforms—reduces cross-study comparability. Third, many included studies focused on specific ethnic populations, potentially missing genetic variants common in other populations, which reduces generalizability. Finally, although robust quality-assessment tools (AXIS, NOS) were employed, differences in study design (cross-sectional vs. cohort vs. case–control) may introduce biases that are difficult to account for.

## Conclusion

The growing field of imaging genetics extends the understanding of the complex interplay between genetic variations and brain structure, function, and connectivity. In this review, FTD was a focus in about 28.5% of the included studies, highlighting its heterogeneity and the variable clinical manifestations arising from different genetic mutations. Advanced imaging techniques have shown how specific genetic factors contribute to neurodegenerative and neurological disorders, potentially enabling earlier diagnosis and personalized interventions. Understanding how genetic variants translate into observable traits is an important aspect of imaging genetics, helping to understand the genotype–phenotype relationship, especially in complex diseases. This can lead to the identification of novel therapeutic targets and personalized treatment strategies. The reviewed articles illustrate a strong genetic influence across diverse neurological conditions, emphasizing the intricate relationship between genotype and neuroanatomy. Future research should expand sample sizes through collaborative consortia like ENIGMA and UK Biobank, adopt standardized imaging protocols, and leverage machine learning for high-dimensional genetic and imaging data. These efforts will accelerate translation to clinical practice, ultimately advancing the goal of personalized neurology and psychiatry.

## Data Availability

No datasets were generated or analysed during the current study.

## Abbreviations

AD: Alzheimer’s disease
AFNI: Analysis of Functional NeuroImages
ANCOVA: Analysis of covariance
ANOVA: Analysis of variance
ANTs: Advanced normalization tools
AUD: Alcohol use disorder
BD: Bipolar disorder
BDNF: Brain-derived neurotrophic factor
bvFTD: Behavioral variant frontotemporal dementia
CNVs: Copy number variants
DTI: Diffusion tensor imaging
FSL: FMRIB Software Library
FTLD: Frontotemporal lobar degeneration
FTD: Frontotemporal dementia
GM: Gray matter
GLM: General linear model
GWAS: Genome-wide association studies
HCP: Human Connectome Project
HSP: Hereditary spastic paraplegia
HNPP: Hereditary neuropathy with liability to pressure palsies
MRI: Magnetic resonance imaging
MDD: Major depressive disorder
NIMH: National Institute of Mental Health
PET: Positron emission tomography
PM: Precision medicine
PRISMA: Preferred Reporting Items for Systematic Reviews and Meta-Analyses
PGC: Psychiatric Genomics Consortium
PPMI: Parkinson’s Progression Markers Initiative
SNP: Single nucleotide polymorphism
SPM: Statistical parametric mapping
TBM: Tensor-based morphometry
VBM: Voxel-based morphometry
WM: White matter

## References

[CR1] Algumaei AH, Algunaid RF, Rushdi MA, Yassine IA (2022) Feature and decision-level fusion for schizophrenia detection based on resting-state fMRI data. PLoS ONE 17(5):e0265300. 10.1371/journal.pone.0265300 35609033 10.1371/journal.pone.0265300 PMC9129055

[CR2] Ameis SH, Szatmari P (2012) Imaging-genetics in autism spectrum disorder: advances, translational impact, and future directions. Front Psychiatry 3:46. 10.3389/fpsyt.2012.00046 22615702 10.3389/fpsyt.2012.00046 PMC3351673

[CR3] Anderson KM, Collins MA, Chin R, Ge T, Rosenberg MD, Holmes AJ (2020) Transcriptional and imaging-genetic association of cortical interneurons, brain function, and schizophrenia risk. Nat Commun 11(1):2889. 10.1038/s41467-020-16710-x 32514083 10.1038/s41467-020-16710-x PMC7280213

[CR4] Ashburner J, Friston KJ (2000) Voxel-based morphometry—the methods. Neuroimage 11(6):805–821. 10.1006/nimg.2000.0582 10860804 10.1006/nimg.2000.0582

[CR5] Ashina M et al (2021) Migraine: disease characterisation, biomarkers, and precision medicine. The Lancet 397(10283):1496–1504. 10.1016/S0140-6736(20)32162-0 10.1016/S0140-6736(20)32162-0 33773610

[CR6] Attarian S, Fatehi F, Rajabally YA, Pareyson D (2020) Hereditary neuropathy with liability to pressure palsies. J Neurol 267(8):2198–2206. 10.1007/s00415-019-09319-8 30989370 10.1007/s00415-019-09319-8

[CR7] Avants BB, Tustison NJ, Song G, Cook PA, Klein A, Gee JC (2011) A reproducible evaluation of ANTs similarity metric performance in brain image registration. Neuroimage 54(3):2033–2044. 10.1016/j.neuroimage.2010.09.025 20851191 10.1016/j.neuroimage.2010.09.025 PMC3065962

[CR8] Baker E, Escott-Price V (2020) Polygenic risk scores in Alzheimer’s disease: current applications and future directions. Front Digit Health 2. 10.3389/fdgth.2020.00014 10.3389/fdgth.2020.00014 PMC8521998 34713027

[CR9] Barch DM et al (2018) Demographic, physical and mental health assessments in the adolescent brain and cognitive development study: rationale and description. Dev Cogn Neurosci 32:55–66. 10.1016/j.dcn.2017.10.010 29113758 10.1016/j.dcn.2017.10.010 PMC5934320

[CR10] Beaulac C et al (2023) Neuroimaging feature extraction using a neural network classifier for imaging genetics. BMC Bioinformatics 24(1):271. 10.1186/s12859-023-05394-x 37391692 10.1186/s12859-023-05394-x PMC10311793

[CR11] Bharthur Sanjay A et al (2022) Characterization of gene expression patterns in mild cognitive impairment using a transcriptomics approach and neuroimaging endophenotypes. Alzheimer’s & Dementia 18(12):2493–2508. 10.1002/alz.12587 10.1002/alz.12587 PMC10078657 35142026

[CR12] Bloem BR, Okun MS, Klein C (2021) Parkinson’s disease. The Lancet 397(10291):2284–2303. 10.1016/S0140-6736(21)00218-X 10.1016/S0140-6736(21)00218-X 33848468

[CR13] Bogdan R et al (2017) Imaging genetics and genomics in psychiatry: a critical review of progress and potential. Biol Psychiatry 82(3):165–175. 10.1016/j.biopsych.2016.12.030 28283186 10.1016/j.biopsych.2016.12.030 PMC5505787

[CR14] Brandt AU et al (2016) Afferent visual pathway affection in patients with PMP22 deletion-related hereditary neuropathy with liability to pressure palsies. PLoS ONE 11(10):e0164617. 10.1371/journal.pone.0164617 27749933 10.1371/journal.pone.0164617 PMC5066968

[CR15] Burciu RG et al (2018) Multimodal neuroimaging and behavioral assessment of α-synuclein polymorphism rs356219 in older adults. Neurobiol Aging 66:32–39. 10.1016/j.neurobiolaging.2018.02.001 29505953 10.1016/j.neurobiolaging.2018.02.001 PMC5924640

[CR16] Carvalho AF, Heilig M, Perez A, Probst C, Rehm J (2019) Alcohol use disorders. The Lancet 394(10200):781–792. 10.1016/S0140-6736(19)31775-1 10.1016/S0140-6736(19)31775-1 31478502

[CR17] Cash DM et al (2018) Patterns of gray matter atrophy in genetic frontotemporal dementia: results from the GENFI study. Neurobiol Aging 62:191–196. 10.1016/j.neurobiolaging.2017.10.008 29172163 10.1016/j.neurobiolaging.2017.10.008 PMC5759893

[CR18] Cataldo-Ramirez CC, Haddad D, Amenta N, Weaver TD (2023) Developing an automated skeletal phenotyping pipeline to leverage biobank-level medical imaging databases. Am J Biol Anthropol 181(3):413–425. 10.1002/ajpa.24736 36974923 10.1002/ajpa.24736

[CR19] Chelladurai A, Narayan DL, Divakarachari PB, Loganathan U (2023) fMRI-based Alzheimer’s disease detection using the SAS method with multi-layer perceptron network. Brain Sci 13(6):893. 10.3390/brainsci13060893 37371371 10.3390/brainsci13060893 PMC10296435

[CR20] Chen J, Liu J, Calhoun VD (2019) Translational potential of neuroimaging genomic analyses to diagnosis and treatment in mental disorders. Proc IEEE 107(5):912–927. 10.1109/JPROC.2019.2913145 10.1109/JPROC.2019.2913145 PMC7015534 32051642

[CR21] Chen JA, Coppola G (2015) Imaging genetics of neuropsychiatric disease. In: Brain Mapping, Elsevier. pp. 1037–1047. 10.1016/B978-0-12-397025-1.00130-5

[CR22] Cousijn H et al (2014) No effect of schizophrenia risk genes MIR137, TCF4, and ZNF804A on macroscopic brain structure. Schizophr Res 159(2–3):329–332. 10.1016/j.schres.2014.08.007 25217366 10.1016/j.schres.2014.08.007 PMC4245712

[CR23] Cox RW (1996) AFNI: software for analysis and visualization of functional magnetic resonance neuroimages. Comput Biomed Res 29(3):162–173. 10.1006/cbmr.1996.0014 8812068 10.1006/cbmr.1996.0014

[CR24] Dalvie S et al (2014) The BDNFp.Val66Met polymorphism, childhood trauma, and brain volumes in adolescents with alcohol abuse. BMC Psychiatry 14(1):328. 10.1186/s12888-014-0328-2 25510982 10.1186/s12888-014-0328-2 PMC4295262

[CR25] Dalvie S, Brooks SJ, Cardenas V, Fein G, Ramesar R, Stein DJ (2017) Genetic variation within *GRIN2B* in adolescents with alcohol use disorder may be associated with larger left posterior cingulate cortex volume. Acta Neuropsychiatr 29(4):252–258. 10.1017/neu.2016.41 27498914 10.1017/neu.2016.41 PMC5478461

[CR26] Dannlowski U et al (2015) NCAN cross-disorder risk variant is associated with limbic gray matter deficits in healthy subjects and major depression. Neuropsychopharmacology 40(11):2510–2516. 10.1038/npp.2015.86 25801500 10.1038/npp.2015.86 PMC4569958

[CR27] de Leeuw CA, Mooij JM, Heskes T, Posthuma D (2015) MAGMA: generalized gene-set analysis of GWAS data. PLoS Comput Biol 11(4):e1004219. 10.1371/journal.pcbi.1004219 25885710 10.1371/journal.pcbi.1004219 PMC4401657

[CR28] Downes MJ, Brennan ML, Williams HC, Dean RS (2016) Development of a critical appraisal tool to assess the quality of cross-sectional studies (AXIS). BMJ Open 6(12):e011458. 10.1136/bmjopen-2016-011458 27932337 10.1136/bmjopen-2016-011458 PMC5168618

[CR29] Du L, Zhang J, Zhao Y, Shang M, Guo L, Han J (2023) inMTSCCA: an integrated multi-task sparse canonical correlation analysis for multi-omic brain imaging genetics. Genomics Proteomics Bioinformatics 21(2):396–413. 10.1016/j.gpb.2023.03.005 37442417 10.1016/j.gpb.2023.03.005 PMC10634656

[CR30] Elliott LT et al (2018) Genome-wide association studies of brain imaging phenotypes in UK Biobank. Nature 562(7726):210–216. 10.1038/s41586-018-0571-7 30305740 10.1038/s41586-018-0571-7 PMC6786974

[CR31] Faber I et al (2018) SPG11 mutations cause widespread white matter and basal ganglia abnormalities, but restricted cortical damage. Neuroimage Clin 19:848–857. 10.1016/j.nicl.2018.05.031 29946510 10.1016/j.nicl.2018.05.031 PMC6008284

[CR32] Fischl B (2012) FreeSurfer. Neuroimage 62(2):774–781. 10.1016/j.neuroimage.2012.01.021 22248573 10.1016/j.neuroimage.2012.01.021 PMC3685476

[CR33] Gao S et al (2021) Comparing empirical kinship derived heritability for imaging genetics traits in the UK biobank and human connectome project. Neuroimage 245:118700. 10.1016/j.neuroimage.2021.118700 34740793 10.1016/j.neuroimage.2021.118700 PMC8771206

[CR34] Glahn DC, et al (2015) Imaging genetics. In: Brain Mapping, Elsevier, 929–932. 10.1016/B978-0-12-397025-1.00112-3

[CR35] Gomar JJ, Conejero-Goldberg C, Huey ED, Davies P, Goldberg TE (2016) Lack of neural compensatory mechanisms of BDNF val66met met carriers and APOE E4 carriers in healthy aging, mild cognitive impairment, and Alzheimer’s disease. Neurobiol Aging 39:165–173. 10.1016/j.neurobiolaging.2015.12.004 26923413 10.1016/j.neurobiolaging.2015.12.004 PMC9969539

[CR36] Grande I, Berk M, Birmaher B, Vieta E (2016) Bipolar disorder. The Lancet 387(10027):1561–1572. 10.1016/S0140-6736(15)00241-X 10.1016/S0140-6736(15)00241-X 26388529

[CR37] Harmouche A, Kövér F, Szukits S, Dóczi T, Bogner P, Tóth A (2023) WebMRI: brain extraction and linear registration in the web browser. Imaging 15(1):31–36. 10.1556/1647.2023.00111

[CR38] He X et al (2021) The morphometry of left cuneus mediating the genetic regulation on working memory. Hum Brain Mapp 42(11):3470–3480. 10.1002/hbm.25446 33939221 10.1002/hbm.25446 PMC8249898

[CR39] Hibar DP et al (2015) Genome-wide interaction analysis reveals replicated epistatic effects on brain structure. Neurobiol Aging 36:S151–S158. 10.1016/j.neurobiolaging.2014.02.033 25264344 10.1016/j.neurobiolaging.2014.02.033 PMC4332874

[CR40] Howie BN, Donnelly P, Marchini J (2009) A flexible and accurate genotype imputation method for the next generation of genome-wide association studies. PLoS Genet 5(6):e1000529. 10.1371/journal.pgen.1000529 19543373 10.1371/journal.pgen.1000529 PMC2689936

[CR41] Huang C-C et al (2016) Effect of Alzheimer’s Disease Risk Variant rs3824968 at SORL1 on regional gray matter volume and age-related interaction in adult lifespan. Sci Rep 6(1):23362. 10.1038/srep23362 26996954 10.1038/srep23362 PMC4800313

[CR42] Jacobs BM et al (2020) Parkinson’s disease determinants, prediction and gene–environment interactions in the UK Biobank. J Neurol Neurosurg Psychiatry 91(10):1046–1054. 10.1136/jnnp-2020-323646 32934108 10.1136/jnnp-2020-323646 PMC7509524

[CR43] Jenkinson M, Beckmann CF, Behrens TEJ, Woolrich MW, Smith SM (2012) FSL. Neuroimage 62(2):782–790. 10.1016/j.neuroimage.2011.09.015 10.1016/j.neuroimage.2011.09.015 21979382

[CR44] Jiang W, King TZ, Turner JA (2019) Imaging genetics towards a refined diagnosis of schizophrenia. Front Psychiatry 10. 10.3389/fpsyt.2019.00494 10.3389/fpsyt.2019.00494 PMC6639711 31354550

[CR45] Juneja A, Rana B, Agrawal RK (2018) A novel fuzzy rough selection of non-linearly extracted features for schizophrenia diagnosis using fMRI. Comput Methods Programs Biomed 155:139–152. 10.1016/j.cmpb.2017.12.001 29512494 10.1016/j.cmpb.2017.12.001

[CR46] Kalmady SV et al (2014) Relationship between interleukin-6 gene polymorphism and hippocampal volume in antipsychotic-naïve schizophrenia: evidence for differential susceptibility? PLoS ONE 9(5):e96021. 10.1371/journal.pone.0096021 24787542 10.1371/journal.pone.0096021 PMC4008499

[CR47] Karkar S et al (2021) Genome-wide haplotype association study in imaging genetics using whole-brain sulcal openings of 16,304 UK Biobank subjects. Eur J Hum Genet 29(9):1424–1437. 10.1038/s41431-021-00827-8 33664500 10.1038/s41431-021-00827-8 PMC8440755

[CR48] Kesby J, Eyles D, McGrath J, Scott J (2018) Dopamine, psychosis and schizophrenia: the widening gap between basic and clinical neuroscience. Transl Psychiatry 8(1):30. 10.1038/s41398-017-0071-9 29382821 10.1038/s41398-017-0071-9 PMC5802623

[CR49] Lee SE et al (2014) Altered network connectivity in frontotemporal dementia with C9orf72 hexanucleotide repeat expansion. Brain 137(11):3047–3060. 10.1093/brain/awu248 25273996 10.1093/brain/awu248 PMC4208465

[CR50] Li W, Lin X, Chen X (2020) Detecting Alzheimer’s disease based on 4D fMRI: an exploration under deep learning framework. Neurocomputing 388:280–287. 10.1016/j.neucom.2020.01.053

[CR51] Liu J et al (2015) Genetic contribution of catechol- *O* -methyltransferase in hippocampal structural and functional changes of female migraine sufferers. Hum Brain Mapp 36(5):1782–1795. 10.1002/hbm.22737 25598522 10.1002/hbm.22737 PMC6869255

[CR52] Lord C et al (2020) Autism spectrum disorder. Nat Rev Dis Primers 6(1):5. 10.1038/s41572-019-0138-4 31949163 10.1038/s41572-019-0138-4 PMC8900942

[CR53] Lorenzi M, Altmann A (2024) Imaging genetics. In: Medical Image Analysis, Elsevier. 549–576. 10.1016/B978-0-12-813657-7.00034-0

[CR54] Luis EO et al (2014) Frontobasal gray matter loss is associated with the TREM2 p. R47H variant. Neurobiol Aging 35(12):2681–2690. 10.1016/j.neurobiolaging.2014.06.007 25027412 10.1016/j.neurobiolaging.2014.06.007 PMC4253600

[CR55] Luis E et al (2016) Neuroimaging correlates of frontotemporal dementia associated with SQSTM1 mutations. J Alzheimer’s Disease 53(1):303–313. 10.3233/JAD-160006 27163810 10.3233/JAD-160006

[CR56] Mahoney CJ et al (2014) Profiles of white matter tract pathology in frontotemporal dementia. Hum Brain Mapp 35(8):4163–4179. 10.1002/hbm.22468 24510641 10.1002/hbm.22468 PMC4312919

[CR57] Marchini J, Howie B, Myers S, McVean G, Donnelly P (2007) A new multipoint method for genome-wide association studies by imputation of genotypes. Nat Genet 39(7):906–913. 10.1038/ng2088 17572673 10.1038/ng2088

[CR58] Marek K et al (2011) The Parkinson progression marker initiative (PPMI). Prog Neurobiol 95(4):629–635. 10.1016/j.pneurobio.2011.09.005 21930184 10.1016/j.pneurobio.2011.09.005 PMC9014725

[CR59] Martin-Brevet S et al (2018) Quantifying the effects of 16p11.2 copy number variants on brain structure: a multisite genetic-first study. Biol Psychiatry 84(4):253–264. 10.1016/j.biopsych.2018.02.1176 29778275 10.1016/j.biopsych.2018.02.1176

[CR60] Marx W et al (2023) Major depressive disorder. Nat Rev Dis Primers 9(1):44. 10.1038/s41572-023-00454-1 37620370 10.1038/s41572-023-00454-1

[CR61] Meyyazhagan A, Orlacchio A (2022) Hereditary spastic paraplegia: an update. Int J Mol Sci 23(3):1697. 10.3390/ijms23031697 35163618 10.3390/ijms23031697 PMC8835766

[CR62] Millman KJ, Brett M (2007) Analysis of functional magnetic resonance imaging in Python. Comput Sci Eng 9(3):52–55. 10.1109/MCSE.2007.46

[CR63] Mueller SG et al (2005) The Alzheimer’s disease neuroimaging initiative. Neuroimaging Clin N Am 15(4):869–877. 10.1016/j.nic.2005.09.008 16443497 10.1016/j.nic.2005.09.008 PMC2376747

[CR64] National Institute of Mental Health. [Online]. Available: https://www.nimh.nih.gov. Accessed 1 Aug 2024

[CR65] Nisar S, Haris M (2023) Neuroimaging genetics approaches to identify new biomarkers for the early diagnosis of autism spectrum disorder. Mol Psychiatry 28(12):4995–5008. 10.1038/s41380-023-02060-9 37069342 10.1038/s41380-023-02060-9 PMC11041805

[CR66] Ota M et al (2016) Effects of ankyrin 3 gene risk variants on brain structures in patients with bipolar disorder and healthy subjects. Psychiatry Clin Neurosci 70(11):498–506. 10.1111/pcn.12431 27488254 10.1111/pcn.12431

[CR67] Owen MJ, Sawa A, Mortensen PB (2016) Schizophrenia. The Lancet 388(10039):86–97. 10.1016/S0140-6736(15)01121-6 10.1016/S0140-6736(15)01121-6 PMC4940219 26777917

[CR68] Pinker K, Chin J, Melsaether AN, Morris EA, Moy L (2018) Precision medicine and radiogenomics in breast cancer: new approaches toward diagnosis and treatment. Radiology 287(3):732–747. 10.1148/radiol.2018172171 29782246 10.1148/radiol.2018172171

[CR69] Psychiatric Genomics Consortium. [Online]. Available: https://www.med.unc.edu/pgc/. Accessed 1 Aug 2024

[CR70] Purcell S et al (2007) PLINK: a tool set for whole-genome association and population-based linkage analyses. Am J Human Genetics 81(3):559–575. 10.1086/519795 17701901 10.1086/519795 PMC1950838

[CR71] Rezende TJR et al (2015) Multimodal MRI-based study in patients with SPG4 mutations. PLoS ONE 10(2):e0117666. 10.1371/journal.pone.0117666 25658484 10.1371/journal.pone.0117666 PMC4320056

[CR72] Saito Y et al (2014) Neural correlate of autistic-like traits and a common allele in the oxytocin receptor gene. Soc Cogn Affect Neurosci 9(10):1443–1450. 10.1093/scan/nst136 23946005 10.1093/scan/nst136 PMC4187262

[CR73] Schumann G et al (2010) The IMAGEN study: reinforcement-related behaviour in normal brain function and psychopathology. Mol Psychiatry 15(12):1128–1139. 10.1038/mp.2010.4 21102431 10.1038/mp.2010.4

[CR74] Sellami L, et al (2018) Distinct neuroanatomical correlates of neuropsychiatric symptoms in the three main forms of genetic frontotemporal dementia in the GENFI cohort. J Alzheimer’s Disease 1–16. 10.3233/JAD-180053 10.3233/JAD-180053 PMC6087430 30010122

[CR75] Shattuck DW, Leahy RM (2002) BrainSuite: an automated cortical surface identification tool. Med Image Anal 6(2):129–142. 10.1016/S1361-8415(02)00054-3 12045000 10.1016/s1361-8415(02)00054-3

[CR76] Shinagawa S et al (2015) Clinicopathological study of patients with *C9ORF72* -associated frontotemporal dementia presenting with delusions. J Geriatr Psychiatry Neurol 28(2):99–107. 10.1177/0891988714554710 25342578 10.1177/0891988714554710 PMC4408221

[CR77] Strenn N, Pålsson E, Liberg B, Landén M, Ekman A (2021) Influence of genetic variations in IL1B on brain region volumes in bipolar patients and controls. Psychiatry Res 296:113606. 10.1016/j.psychres.2020.113606 33348197 10.1016/j.psychres.2020.113606

[CR78] Sudlow C et al (2015) UK Biobank: an open access resource for identifying the causes of a wide range of complex diseases of middle and old age. PLoS Med 12(3):e1001779. 10.1371/journal.pmed.1001779 25826379 10.1371/journal.pmed.1001779 PMC4380465

[CR79] Teipel SJ et al (2007) Multivariate deformation-based analysis of brain atrophy to predict Alzheimer’s disease in mild cognitive impairment. Neuroimage 38(1):13–24. 10.1016/j.neuroimage.2007.07.008 17827035 10.1016/j.neuroimage.2007.07.008

[CR80] Thompson PM et al (2014) The ENIGMA consortium: large-scale collaborative analyses of neuroimaging and genetic data. Brain Imaging Behav 8(2):153–182. 10.1007/s11682-013-9269-5 24399358 10.1007/s11682-013-9269-5 PMC4008818

[CR81] Ulep MG, Saraon SK, McLea S (2018) Alzheimer disease. J Nurse Practitioners 14(3):129–135. 10.1016/j.nurpra.2017.10.014

[CR82] Van Essen DC et al (2012) The human connectome project: a data acquisition perspective. Neuroimage 62(4):2222–2231. 10.1016/j.neuroimage.2012.02.018 22366334 10.1016/j.neuroimage.2012.02.018 PMC3606888

[CR83] Viar-Hernández D, Rodriguez-Vila B, Gil-Correa M, Malpica N, Torrado-Carvajal A (2024) A case study of medical image software evolution and its impact in the medical imaging community. Heliyon 10(5):e26408. 10.1016/j.heliyon.2024.e26408 38434256 10.1016/j.heliyon.2024.e26408 PMC10907511

[CR84] Vilas D et al (2016) Nigral and striatal connectivity alterations in asymptomatic *LRRK2* mutation carriers: a magnetic resonance imaging study. Mov Disord 31(12):1820–1828. 10.1002/mds.26799 27653520 10.1002/mds.26799

[CR85] Wegdan A, Saad A, Ahmed S, Alsharif MHK, Elfaki A (2023) Cortical thickness and cortical volume measurements of the cingulate gyrus in Sudanese young adult using BrainSuite. New Armenian Med J 1, 17(2023):70–76. 10.56936/18290825-2023.17.70-76

[CR86] Wells G, et al (n.d.) The Newcastle-Ottawa scale (NOS) for assessing the quality of nonrandomized studies in meta- analysis. [Online]. Available: http://www.ohri.ca/programs/clinical_epidemiology/oxford.asp. Accessed 21 Feb 2025

[CR87] Whitwell JL et al (2015) Brain atrophy over time in genetic and sporadic frontotemporal dementia: a study of 198 serial magnetic resonance images. Eur J Neurol 22(5):745–752. 10.1111/ene.12675 25683866 10.1111/ene.12675 PMC4390434

[CR88] Yang J, Lee SH, Goddard ME, Visscher PM (2011) GCTA: a tool for genome-wide complex trait analysis. The American Journal of Human Genetics 88(1):76–82. 10.1016/j.ajhg.2010.11.011 21167468 10.1016/j.ajhg.2010.11.011 PMC3014363

[CR89] Yang W, Singla R, Maheshwari O, Fontaine CJ, Gil-Mohapel J (2022) Alcohol use disorder: neurobiology and therapeutics. Biomedicines 10(5):1192. 10.3390/biomedicines10051192 35625928 10.3390/biomedicines10051192 PMC9139063

[CR90] Yao X et al (2020) Regional imaging genetic enrichment analysis. Bioinformatics 36(8):2554–2560. 10.1093/bioinformatics/btz948 31860065 10.1093/bioinformatics/btz948 PMC7178438

[CR91] Yokoyama JS et al (2015) The 5-HTTLPR variant in the serotonin transporter gene modifies degeneration of brain regions important for emotion in behavioral variant frontotemporal dementia. Neuroimage Clin 9:283–290. 10.1016/j.nicl.2015.07.017 26509115 10.1016/j.nicl.2015.07.017 PMC4576414

[CR92] Zhang J, Ma Z, Yang Y, Guo L, Du L (2024) Modeling genotype–protein interaction and correlation for Alzheimer’s disease: a multi-omics imaging genetics study. Brief Bioinform 25(2):bbae038. 10.1093/bib/bbae038 38348747 10.1093/bib/bbae038 PMC10939371

[CR93] Zhang L, Pan Y, Huang G, Liang Z, Li L, Zhang Z (2021) An imaging genetics study based on brain-wide genome-wide association for identifying quantitative trait loci related to pain sensitivity. In: 2021 14th International Congress on Image and Signal Processing, BioMedical Engineering and Informatics (CISP-BMEI), IEEE 1–4. 10.1109/CISP-BMEI53629.2021.9624411

